# Neurogenetic asymmetries in the catshark developing habenulae: mechanistic and evolutionary implications

**DOI:** 10.1038/s41598-018-22851-3

**Published:** 2018-03-15

**Authors:** Ronan Lagadec, Maxence Lanoizelet, Nuria Sánchez-Farías, Fanny Hérard, Arnaud Menuet, Hélène Mayeur, Bernard Billoud, Isabel Rodriguez-Moldes, Eva Candal, Sylvie Mazan

**Affiliations:** 1 CNRS, Sorbonne Université, Biologie Intégrative des Organismes Marins, UMR7232, F66650 Banyuls-sur-Mer, France; 2 Departmento de Bioloxía Funcional, Universidade de Santiago de Compostela, Santiago de Compostela, E 15782 Spain; 3 CNRS Université d’Orléans, UMR7355, F45071 Orléans, France; 4 CNRS, Sorbonne Université, Station Biologique, FR2424, F29680 Roscoff, France; 5 CNRS, Sorbonne Université, Laboratoire de Biologie Intégrative des Modèles Marins, UMR8227, F29680 Roscoff, France

## Abstract

Analysis of the establishment of epithalamic asymmetry in two non-conventional model organisms, a cartilaginous fish and a lamprey, has suggested that an essential role of Nodal signalling, likely to be ancestral in vertebrates, may have been largely lost in zebrafish. In order to decipher the cellular mechanisms underlying this divergence, we have characterised neurogenetic asymmetries during habenular development in the catshark *Scyliorhinus canicula* and addressed the mechanism involved in this process. As in zebrafish, neuronal differentiation starts earlier on the left side in the catshark habenulae, suggesting the conservation of a temporal regulation of neurogenesis. At later stages, marked, Alk4/5/7 dependent, size asymmetries having no clear counterparts in zebrafish also develop in neural progenitor territories, with a larger size of the proliferative, pseudostratified neuroepithelium, in the right habenula relative to the left one, but a higher cell number on the left of a more lateral, later formed population of neural progenitors. These data show that mechanisms resulting in an asymmetric, preferential maintenance of neural progenitors act both in the left and the right habenulae, on different cell populations. Such mechanisms may provide a substrate for quantitative variations accounting for the variability in size and laterality of habenular asymmetries across vertebrates.

## Introduction

Epithalamic asymmetries provide an interesting model system to address the molecular and cellular bases for morphological variations across vertebrates. Asymmetries between the left and right habenulae, bilateral epithalamic structures, are widespread across vertebrates including humans^1,2^, but with highly variable degrees in their magnitude. Analyses of the mechanisms involved in their formation in a teleost fish, the zebrafish^1,3,4^, an agnathan, the lamprey *P. marinus*, and a cartilaginous fish, the catshark *S. canicula*^5^, have highlighted a remarkable divergence between these three species. In the zebrafish, the formation of habenular asymmetries is strictly dependent on a secreted signal from the parapineal, a small group of neurons, which migrate from the dorsal midline to the left of the epithalamus and play an instructive role in the elaboration of left habenular identity^6^. Although a left sided Nodal activity is the first asymmetry observed in the developing diencephalon, inactivation of the pathway does not abolish habenular asymmetry formation and has only minor effects on their elaboration^3,6,7^. The catshark and lamprey share an early left restricted diencephalic Nodal activity with the zebrafish. However, no evidence for a conservation of a parapineal dependent mechanism can be found in these species and a pharmacological treatment abolishing the early window of Nodal activity also results in the loss of later habenular asymmetries^5^.

Comparisons between the cellular mechanisms controlling habenular asymmetry formation in the catshark and lamprey with those dependent on the parapineal in the zebrafish, are essential to understand the molecular basis for this mechanistic divergence. In the latter, habenular asymmetries essentially consist in different size ratios between two domains of distinct neuronal identities (dHbm, or dorsal medial habenula, and dHbl, or dorsal lateral habenula). The cellular mechanism underlying this difference between the left and the right sides relies on a Wnt and parapineal dependent asymmetric regulation of the corresponding cell fate choices^8,9^. Despite the absence of major effects of Nodal inactivation on habenular asymmetry formation, both direct and indirect roles of the pathway have also been demonstrated in the zebrafish developing epithalamus. Neurogenesis is subject to an asymmetric temporal regulation during habenular development, with a Nodal dependent and parapineal independent delay of the onset of neuronal differentiation in the right habenula relative to the left one^9,10^. The contribution of this process to the establishment of final habenular asymmetries in the zebrafish remains unclear but this observation has led to the suggestion that an ancestral role of Nodal in epithalamic asymmetry formation may involve an asymmetric regulation of neurogenesis^5^. Nodal also regulates parapineal cell number, thus indirectly impacting the elaboration of habenular asymmetries^11^. Here, we show that neurogenesis and the maintenance of neural progenitors are subject to asymmetric regulations in the developing catshark habenulae and that these asymmetries are lost following a pharmacological treatment known to inhibit the early left restricted diencephalic Nodal activity. Unexpectedly, the effects observed differ depending on the progenitor pool considered, neural progenitors appearing preferentially maintained on the left side in a lateral territory, but on the right one in a more medial territory. These data shed light on asymmetrically regulated cellular processes in the catshark developing habenulae and prompt novel hypotheses to account for the variability of habenular size asymmetries across vertebrates.

## Results

### Early neuronal differentiation is asymmetric in the developing catshark habenulae

**Figure 1:**
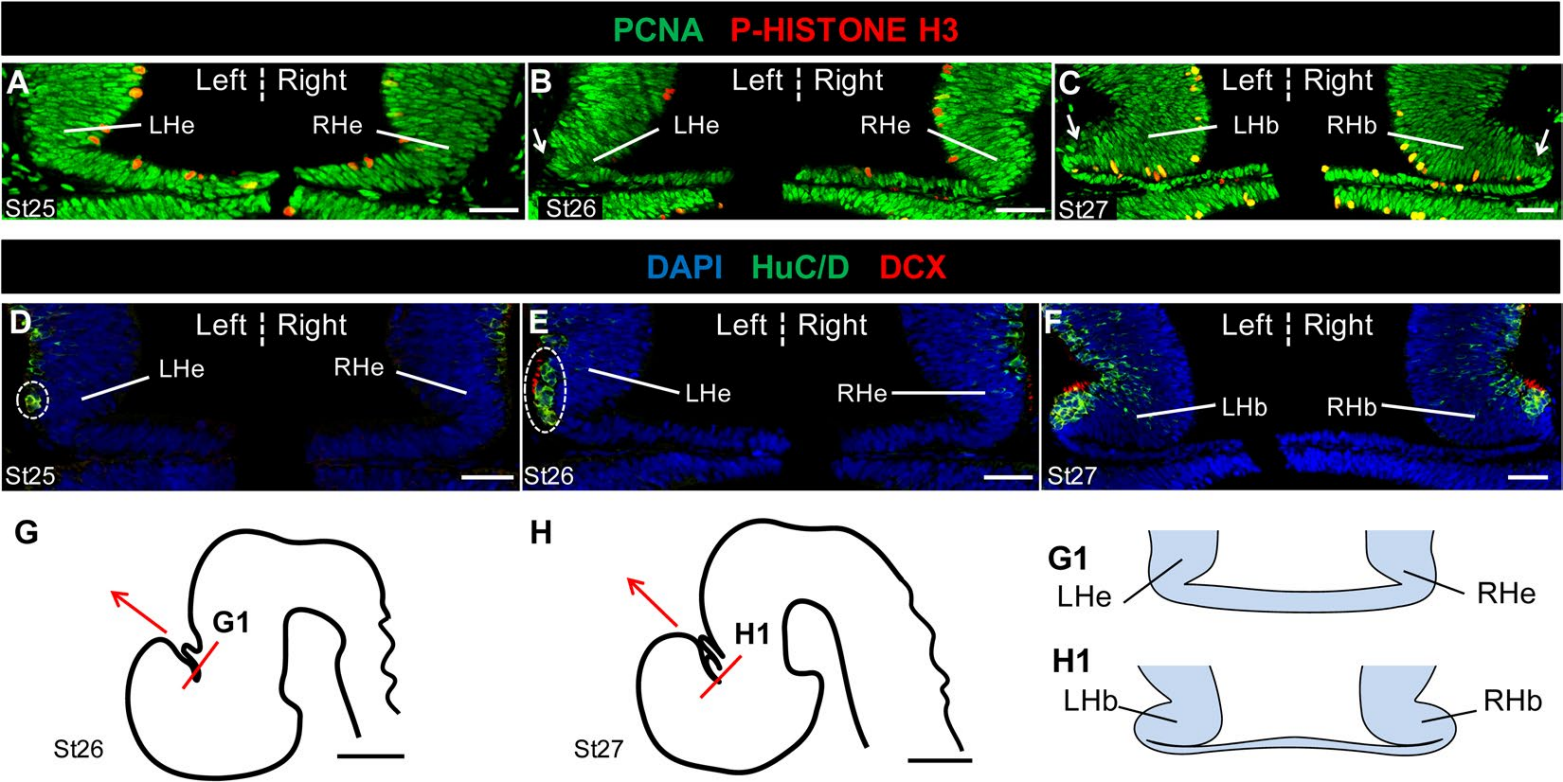
Early neuronal differentiation is asymmetric in the developing catshark habenulae. Transverse sections of catshark habenulae following IHC using antibodies against PCNA (**A**–**C**, green), PH3 (**A**–**C**, red), HuC/D (**D**–**F**, green) and DCX (**D**–**F**, red). DAPI stained nuclei are shown in blue in (**D**–**F**). (**G**,**H**) are schemes of the catshark embryonic brain (lateral views; **G**, stage 26; **H**, stage 27), with a red line indicating the location and plane of the sections analysed and a red arrow showing the antero-posterior axis (arrow pointing towards anterior). (G1,H1) show schemes of the habenula sections at the levels respectively indicated by the red line in (**G**,**H**). Stages are as indicated: (**A**,**D**), stage 25; (**B**,**E**), stage 26; (**C**,**F**) stage 27. White arrows in (**B**,**C**) point to lateral PCNA negative territories of habenular evaginations. Dotted circles in (**D**,**E**) highlight asymmetric HuC/D positive territories. Abbreviations: LHe and RHe, left and right habenular evaginations; LHb and RHb, left and right habenulae. Scale bars = 50 μm in (**A**–**F**), 500 μm in (**G**,**H**).

Catshark habenulae first become visible at stage 25 as two lateral diencephalic evaginations, which expand laterally at subsequent stages (Fig. 1A–F; Fig. S1A–D). To characterise the timing of neurogenesis in the catshark, we first analysed the distribution of proliferation markers, PCNA (proliferating cell nuclear antigen) and PH3 (phospho-histone H3), and neuronal differentiation markers, HuC/D (Hu antigens) and DCX (doublecortin), respectively expressed in post-mitotic and migrating neurons^12,13^. At stages 25 to 27, most cells in the habenular evaginations are positive for PCNA, except for a small number at the lateral tip of the organ from stage 26 onwards (Fig. 1A–C). Expression of HuC/D is first observed in the left habenular evagination at stage 25. No signal is present on the right at this stage (Fig. 1D). At stage 26, HuC/D and DCX expressions overlap in a broader lateral territory of the left habenula, while only a few expressing cells become visible on the right (Fig. 1E). At stage 27, these asymmetries are no longer detectable, HuC/D and DCX positive territories appear established in both developing habenulae, without obvious size difference (Fig. 1F). These data highlight an early, transient asymmetry of neuronal differentiation in the developing catshark habenulae, reminiscent of the unilateral, left presence of habenular neurons observed at the onset of neuronal differentiation in the zebrafish^9,10^.

### Neural progenitors are preferentially maintained in the left, versus right, lateral ventricular zone (LVZ)

**Figure 2:**
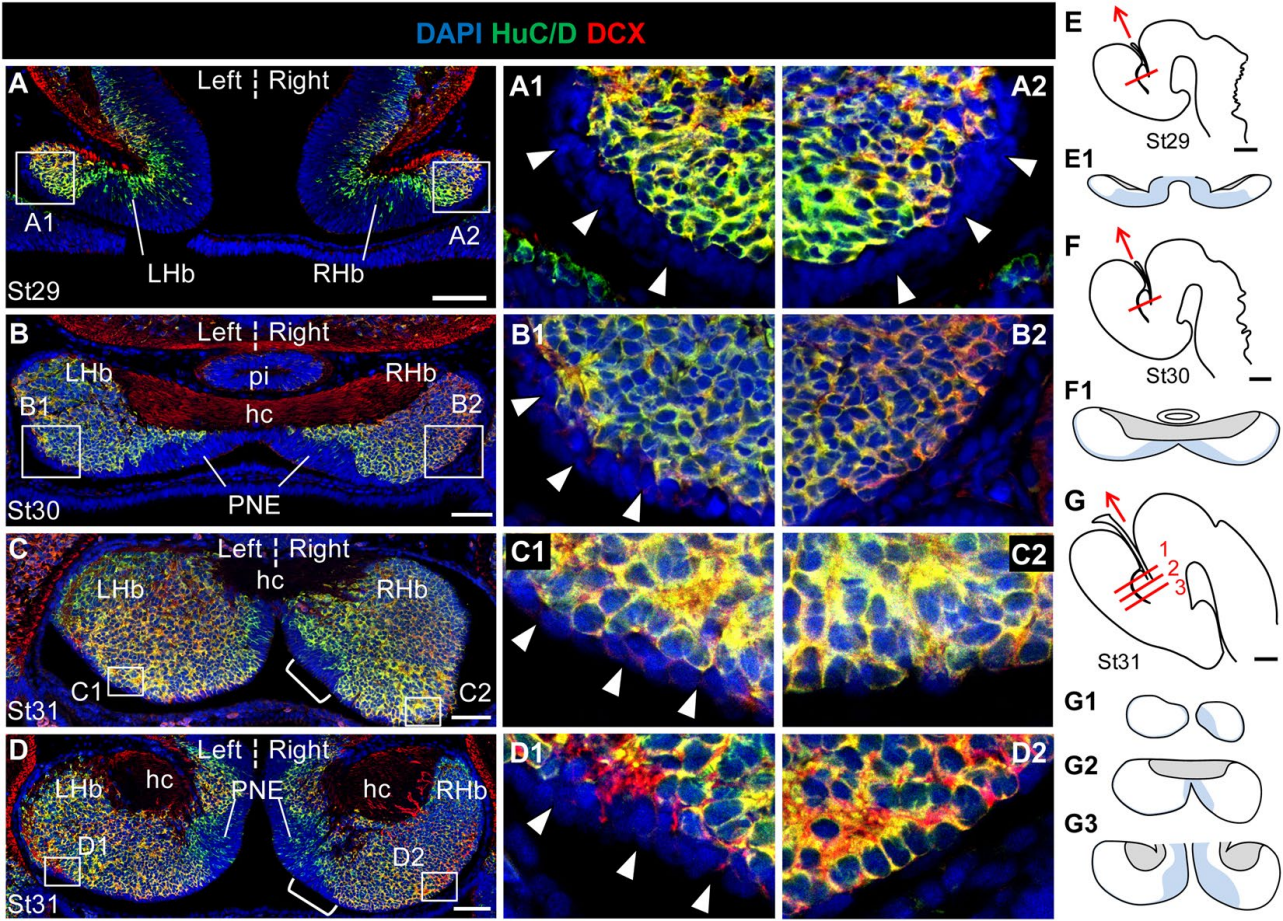
Neuronal differentiation in the LVZ is asymmetric. Transverse sections of catshark habenulae following IHC using antibodies against HuC/D (green) and DCX (red) at stages 29 (**A**), 30 (**B**) and 31 (**C**,**D**). DAPI stained nuclei are shown in blue. (**C**) and (**D**) are located at medial and posterior levels of the habenulae, respectively. (**E**–**G**) are schemes of the catshark embryonic brain (lateral views; **E**, stage 29; **F**, stage30; **G**, stage 31), with a red line indicating the location and plane of the sections analysed and a red arrow showing the antero-posterior axis (arrow pointing towards anterior). (**E1**,**F1**,**G1-3**) show schemes of the habenula sections at the levels respectively indicated by the red line in (**E**,**F**,**G**), with the proliferative neuroepithelium shaded in light blue. (**G1**), (**G2**) and (**G3**) respectively correspond to sections at anterior, medial and posterior levels, labelled 1, 2 and 3 in (**G**). (**A1**,**A2**), (**B1**,**B2**), (**C1**,**C2**), and (**D1**,**D2**) show higher magnifications of LVZ territories boxed in (**A**), (**B**), (**C**) and (**D**), respectively. Brackets in (**C**,**D**) delineate a size extension of the PNE on the right compared to the left. White arrowheads in (**A1**,**A2**,**B1**,**C1**,**D1**) point to LVZ HuC/D and DCX negative cells that are maintained on the left but not the right at stages 30–31. Abbreviations: LHb and RHb, left and right habenulae; hc, habenular commissure; pi, pineal stalk; PNE, pseudo-stratified neuroepithelium. Scale bars = 100 μm in (**A**–**D**), 500 μm in (**E**–**G**).

**Figure 3:**
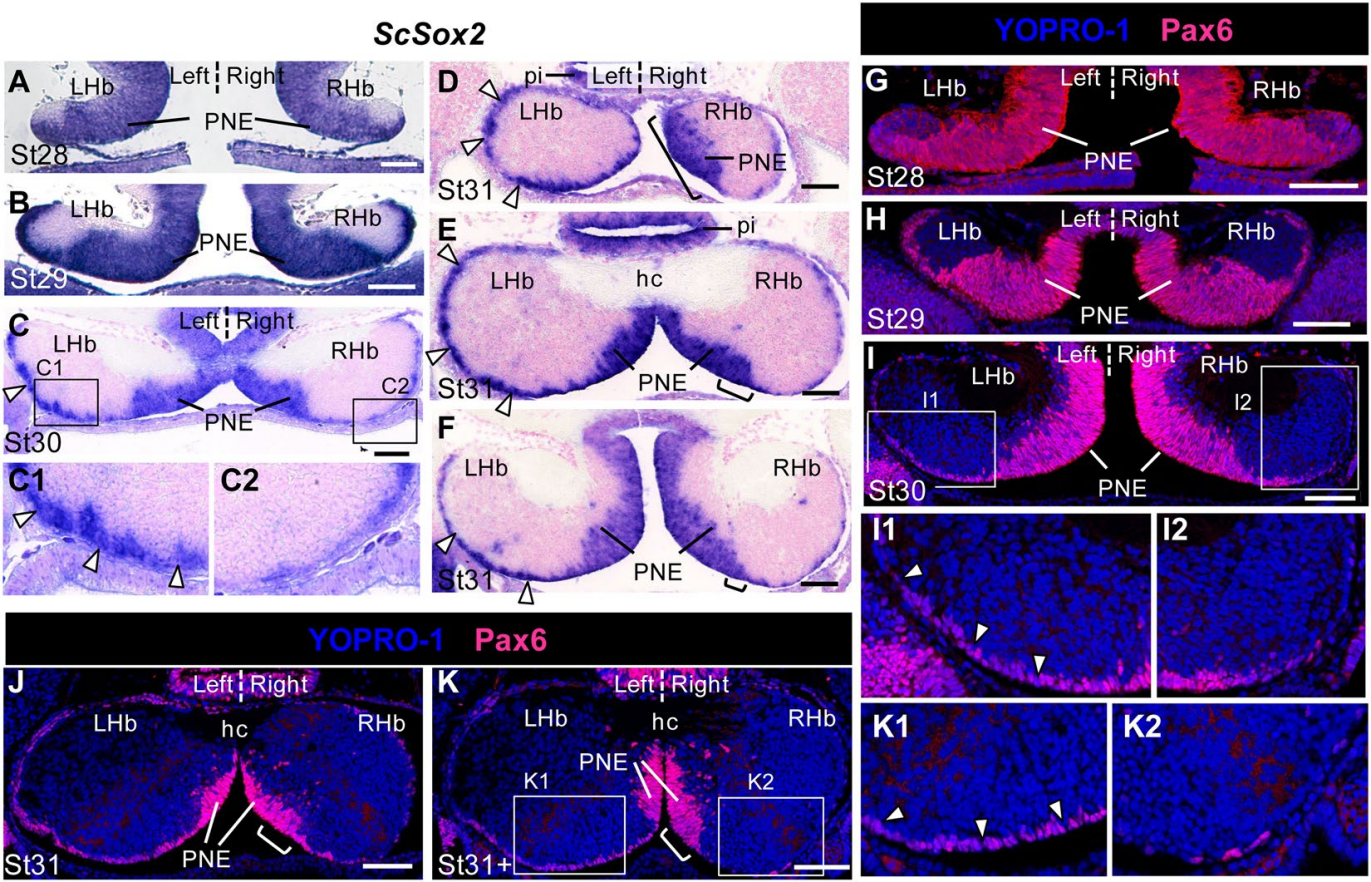
Asymmetric maintenance of neural progenitors in the LVZ and the PNE. Transverse sections of catshark habenulae following ISH using a *ScSox2* probe (**A**–**F**) and IHC using antibodies against Pax6 (red) with a YOPRO-1 nuclei staining in blue (**G**–**K**). Stages are as indicated: (**A**,**G**), stage 28; (**B**–**H**), stage 29; (**C,I**), stage 30; (**D**–**F**,**J**), stage 31; (**J**), late stage 31 (**K**). Section levels and planes are as depicted in Figs 1 and 2, with (**D**), (**E**,**J**,**K**) and (**F**) being respectively located at anterior, medial and posterior levels of the habenulae, as defined in Fig. 2G,G1-3. (**C1**,**C2**), (**I1**,**I2**) and (**K1**,**K2**) show higher magnifications of LVZ territories boxed in (**C**), (**I**) and (**K**).Brackets in (**D**–**F**,**J**,**K**) show the size extension of the PNE on the right relative to the left. Arrowheads in (**C**,**C1**,**D**,**E**,**F**,**I1**,**K1**) point to left LVZ territories showing stronger *ScSox2* expressions (**C**,**C1**,**D**,**E**,**F**) or more Pax6 expressing cells (**I1**,**K1**) than their right counterparts (**C2**,**I2**,**K2**). Abbreviations are as in Fig. 2. Scale bars = 100 μm.

We next extended analysis of HuC/D and DCX expression to stages 29 to 31, when marked size asymmetries appear between the left and right habenulae (Fig. 2). From stage 27 onwards, two territories exhibiting distinct cell organisations are observed on histological sections: a medial ventricular one, showing a typical pseudostratified neuroepithelium cell organisation (PNE), and a lateral one, containing dispersed, round shaped cells, which expands as development proceeds (Fig. S1). At all stages analysed, expression of HuC/D and DCX is mainly restricted to the latter, except for a few HuC/D scattered cells in the PNE (Fig. 2A–D). Higher magnification views show that starting from stage 29, the labelling also excludes a thin layer of cells lining the ventricular zone laterally to the PNE, referred to as lateral ventricular zone (LVZ: Fig. 2A1,A2). No evidence for HuC/D or DCX positive cells is observed at this level either on the left or on the right at stage 29 (Fig. 2A,A1,A2). In contrast, at stage 30, discrete HuC/D and DCX cell clusters appear on the right, while the left LVZ remains unlabelled (Fig. 2B,B1,B2). At stage 31, most cells are HuC/D and DCX immunoreactive in the right LVZ, while they remain largely unlabelled in the left LVZ (Fig. 2C,C1,C2,D,D1,D2). These data suggest that neuronal differentiation in the LVZ is differentially regulated between the left and right habenulae. In order to test whether LVZ neural progenitors may be differentially maintained between the left and right habenulae, we analysed expression of *ScSox2*, the catshark orthologue of *Sox2*, a maintenance factor of neural progenitor identity^14,15^, and Pax6, a marker of neural progenitors^16^, from stages 28 to 31 (Fig. 3). A strong, bilateral signal is observed for both markers in the LVZ at all stages studied (Fig. 3A–I). *ScSox2* and Pax6 are strongly expressed in the LVZ when it becomes visible at stage 29 (Fig. 3B,H). At stage 30, while the LVZ *ScSox2* signal is maintained as a continuous and strongly labelled territory on the left, it becomes patchy, with a fainter intensity on the right (Fig. 3C,C1,C2). This difference is maintained at stage 31 (Fig. 3D–F). Similarly, at stages 30–31, Pax6 positive cells form a dense ventricular layer in the left LVZ, while they appear dispersed along the right LVZ, only few cells remaining at late stage 31 (Fig. 3I,I1,I2,J,K,K1,K2). These data support the conclusion that habenular neural progenitors are preferentially maintained in the left, versus right LVZ.

### The PNE exhibits a larger size in the right habenula than in the left one at stage 31

**Figure 4:**
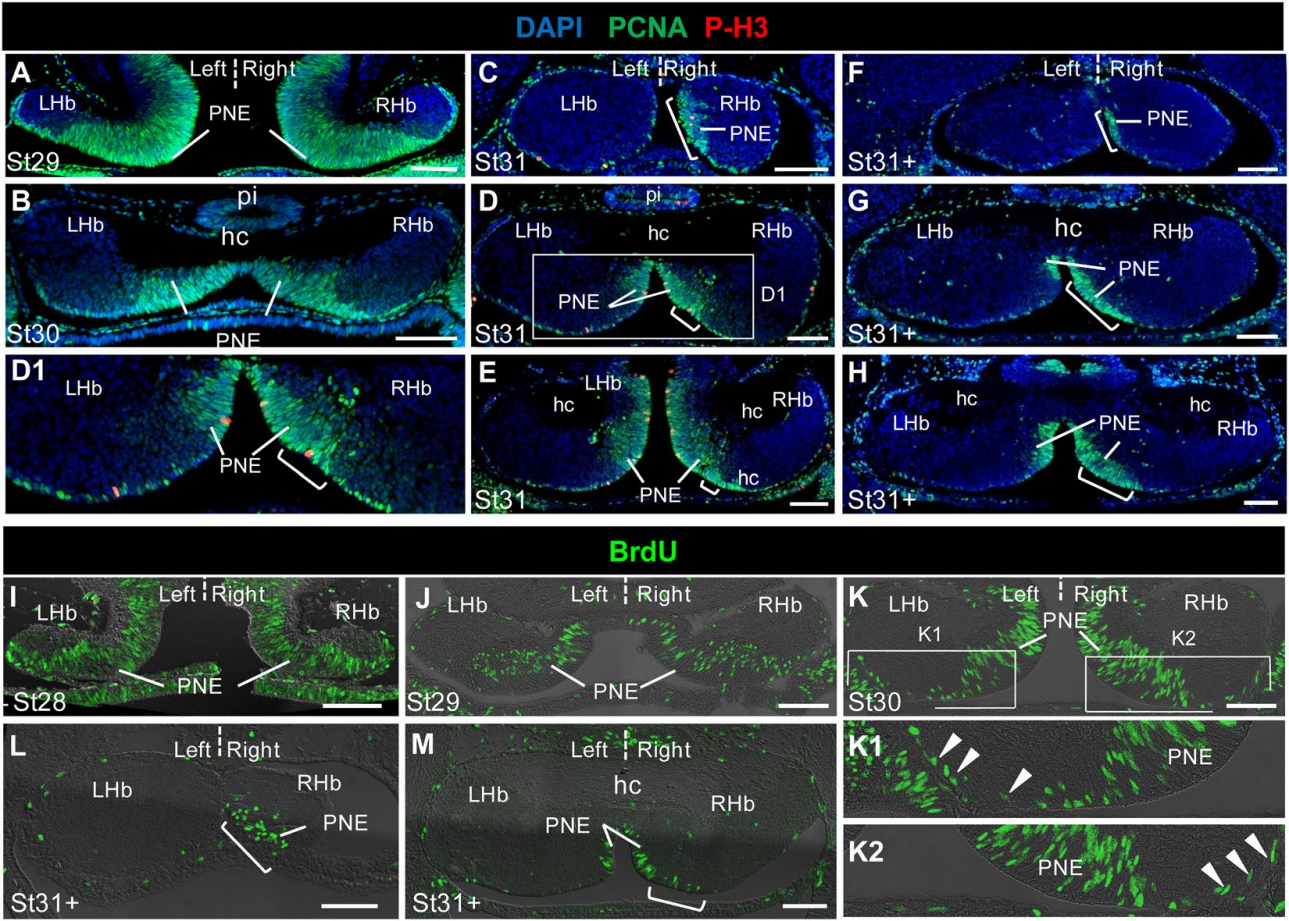
Cell proliferation asymmetries in the LVZ and the PNE. Transverse sections of catshark habenulae following IHC using antibodies against PCNA (green) and PH3 (red) with a DAPI nuclei staining in blue (**A**–**H**) or following a BrdU pulse labelling (**I**–**M**). Stages are as indicated: (**I**), stage 28; (**A**,**J**), stage 29; (**B**,**K**), stage 30; (**C**–**E**) stage 31; (**F**–**H**,**L**,**M**), late stage 31. (**C**,**F**,**L**), (**D**,**G**,**M**) and (**E**,**H**) are respectively located at anterior, medial and posterior levels of the habenulae,as defiend in Fig. 2G,G1-3. (**K1**,**K2**) show higher magnifications of left and right LVZ territories boxed in (**K**). (**D1**) shows a higher magnification of the PNE territories boxed in (**D**). Brackets in (**C**-**H**,**L**,**M**) show the size extension of the PNE on the right relative to the left. Arrowheads in (**K1**,**K2**) point to BrdU labeled cells in the LVZ. Abbreviations are as in Fig. 2. Scale bars = 100 μm.

From stages 29 to late 31, the PNE becomes restricted to the medial part of the habenulae, while differentiating HuC/D and DCX positive territories expand in lateral regions (Fig. 2A–D; Fig. S1C,D,F,G). *ScSox2*, PCNA and PAX6 are strongly expressed in this territory at these stages, supporting its proliferative neuroepithelial nature (Fig. 3A–K; Fig. 4A–H). From stages 27 onwards, the PNE also expresses *ScNgn2* and *ScNeuroD1*, the catshark orthologues of the proneural genes *Ngn2* and *NeuroD1*^17^. Expression of the latter is restricted to the peripheral PNE, suggesting that the two territories may correspond to different stages of the neurogenic process (Fig. S2A–J). No asymmetry in the *ScSox2*, *ScNgn2*, Pax6 and PCNA expressing PNE territories is observed from stages 26 to 30 (Fig. 3A–C,G–I; Fig. 4A,B; Fig. S2B–D). In contrast, at stages 31-31+, expressions of *ScSox2* (Fig. 3D–F; see also Fig. S5), *ScNgn2* (Fig. S2E), Pax6 (Fig. 3J,K), PCNA (Fig. 4C–E,D1; Fig. S3F–H) extend further laterally in the PNE on the right than the left. This asymmetry is most obvious in the anterior habenulae (Fig. 3D,E; Fig. 4C,D; Fig. S5) and maintained at late stage 31 (Fig. 3K; Fig. 4F–H), as assessed by PCNA and Pax6 labelling. At stage 32, PCNA immunoreactivity has withdrawn from the ventricular zone except for a few scattered cells in its medial part (Fig. S2M). This dynamic expression profile is consistent with an asymmetric regulation of the maintenance of PNE neural progenitors, which persist in a broader territory in the left habenula than in the right one at stage 31-31+.

### Cell proliferation is asymmetrically regulated in the LVZ

**Figure 5:**
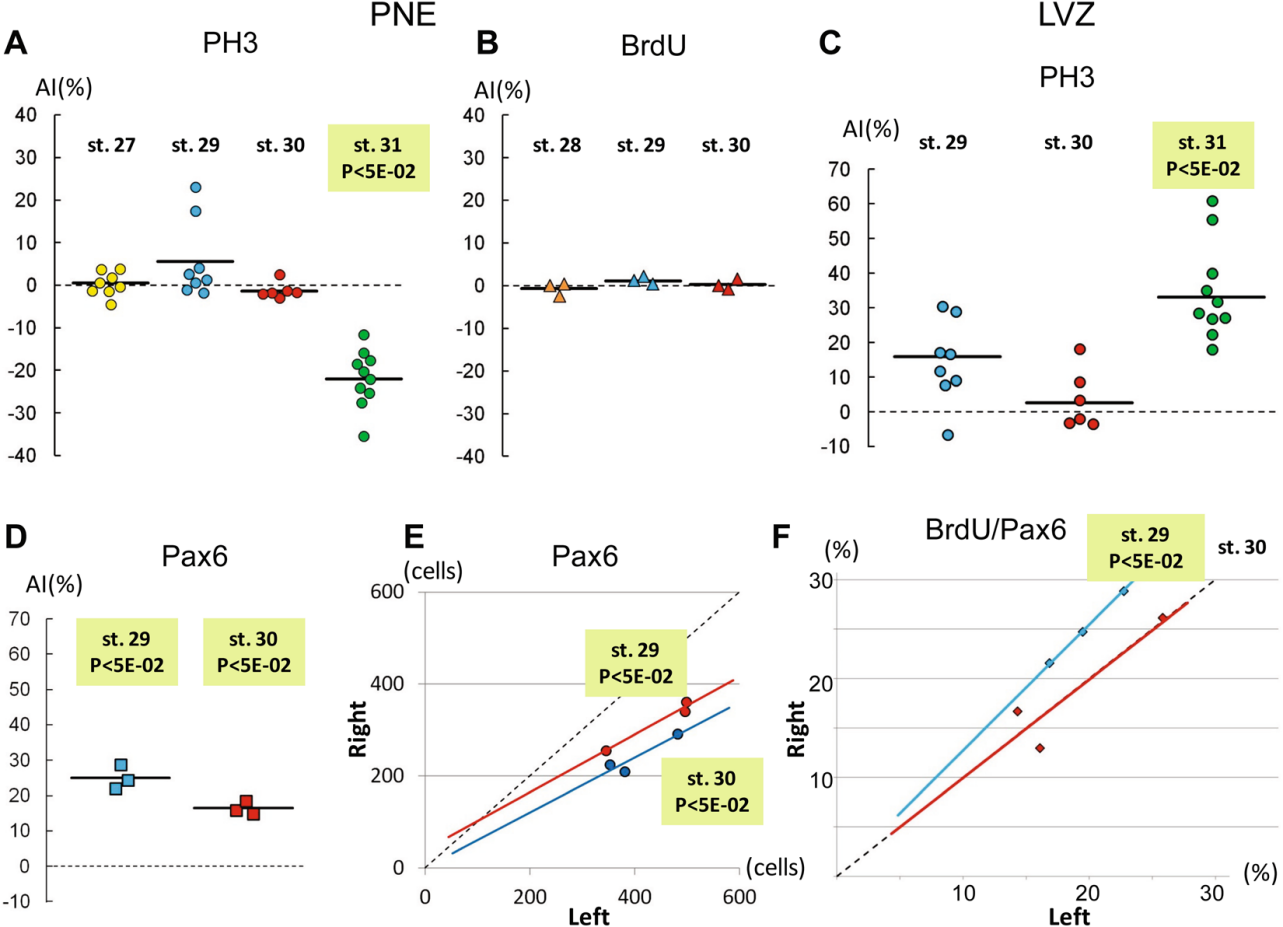
Quantified analysis of proliferation asymmetries in the LVZ and PNE. (**A**–**D**) Plots showing asymmetry indexes (**AI**) in the PNE (**A**,**B**) and in the LVZ (**C**,**D**), with regard to counts of PH3 (**A**,**C**), BrdU (**B**) and Pax6 (**D**) positive cells at stages 27 to 31. Dots in (A,C), triangles in (**B**) and squares in (**D**) correspond to AIs obtained for individual embryos at stages 27 (yellow), 28 (orange), 29 (blue), 30 (red) and 31 (green). For each group of embryos analysed (same marker, same stage, same zone), horizontal black lines show the median AI value. (**E**,**F**) Plots showing Pax6 positive cell counts (**E**) and ratios (in %) of BrdU to Pax6 positive cell counts (**F**) in the right and in the left LVZ at stages 29 and 30. Blue and red dots correspond to individual embryos respectively at stage 29 and at stage 30. Best-fit regression lines (blue at stage 29, red at stage 30) are shown, together with the symmetry reference (dotted black line). Light green shading in (**A**,**C**–**F**) with the indication (P < 5E-02) highlight embryo groups exhibiting a significant asymmetry in the distribution of cell counts between the left and the right, based on paired Student’s t-tests on AIs (**A**,**C**,**D**) and ratios of BrdU to Pax6 cell counts (**F**). In (**E**), this indication refers to X^2^ tests conducted on all individual embryos analysed. Abbreviation used: P, p-value.

In order to address whether cell proliferation may be asymmetrically regulated in the PNE or LVZ, we compared the total number of neural progenitors and relative proportion of dividing cells in each of these territories between the left and the right. The statistical significance of differences between the left and the right habenulae was assessed at the level of individual embryos or at the level of the group of embryos analysed, respectively using Χ² tests on cell counts or paired Student’s t-tests on the mean difference of the variable analysed (cell counts or ratios). Both tests were used when applicable. In the PNE, there is no evidence for a difference in cell density between the left and the right from stage 27 to 31 (Χ² tests on cell counts from individual embryos; n > 46; p-values between 0.42 and 0.92; Table S1). The count distribution of PH3 positive, mitotic cells also exhibits no statistically significant difference between the left and the right PNE at stages 27 (n = 8), 29 (n = 8) and 30 (n = 6) (paired Student’s t-tests; p-values comprised between 0.1 and 0.6). Mean AI (asymmetry index) values do not exceed +/−10% at these stages (Fig. 5A; Tables S2 and S3). Similarly, no significant difference in counts of BrdU (5-bromo-2-deoxyuridine) positive cells can be detected between the left and right PNE of the embryos analysed at stages 28 (n = 3), 29 (n = 3) and 30 (n = 3) following BrdU pulse labelling (Χ² tests on cell counts from individual embryos; n > 500; p-values comprised between 0.399 and 0.976; AI values comprised between −0.7 and 1.7%; Fig. 5B; Table S4). In contrast, at stage 31, the count distribution of PH3 positive cells in the PNE appears significantly asymmetric (n = 10; paired Student’s t-test; p-value = 4.0810E-04; Fig. 5A; Table S3), with higher values on the right than on the left in all embryos tested (AI values ranging from −12% to −36%) and with statistically significant asymmetries in 7 out of 10 embryos analysed (Χ² tests on cell counts from individual embryos; Table S2). In order to test whether the PNE size, larger on the right than on the left at stage 31, could account for this asymmetry, we directly counted PH3 positive cells in the right PNE expansion for three embryos (Table S5). No significant difference is detected between the left and the right sides if this expansion is excluded from the counts, in either one of the three embryos analysed (Χ² tests on cell counts from individual embryos; n > 52; p-values comprised between 0.785 and 0.966; Table S5). In the absence of overt difference in cell density between the left PNE and the right one, this suggests that the difference in PH3 counts observed at stage 31 is not related to an asymmetry in proliferation rate and only reflects the larger PNE size on the right than on the left. In the LVZ, the count distribution of PH3 positive cells at stage 31 highlights a significant asymmetry between the right and the left sides (n = 10; paired Student’s t-test; p-value = 4.16E-05), with fewer labelled cells on the right than on the left in all embryos analysed and AI values comprised between 18 and 61% (Fig. 5C; Tables S2 and S3). An asymmetric distribution of cell counts is also detected at stage 29, albeit with lower AIs including positive and negative values (AIs comprised between −7% and 31%) and a lower statistical support (n = 8; Student’s t-test; p-value = 1.5950E-02), but not at stage 30 (n = 6; Student’s t-test; p-value = 0.4017) (Fig. 5C; Tables S2 and S3). In view of the low number of PH3 labelled cells in the LVZ and of the absence of significant asymmetries for most individual embryos analysed with regard to this parameter (X^2^ test p-values > 5E-02 except for one embryo; Table S2), we conducted BrdU incorporation experiments in order to further assess cell proliferation asymmetries in this cell population. Pax6 immuno-detection was also used in this analysis to estimate the number of neural progenitors in the left and right LVZ and normalise counts of BrdU labelled cells. This analysis was focused on stages 29 and 30, when no overt size asymmetry is detected between the left and right habenulae. A significantly higher number of Pax6 expressing neural progenitors is observed in the left LVZ than in the right one in all embryos analysed not only at stage 30, in line with the broader *ScSox2* and Pax6 expression territories observed (see above; Fig. 3), but also at stage 29 (Χ² tests on cell counts from individual embryos; n > 200; p-values comprised between 2.582E-12 and 2.83E-04; Fig. 5E; Table S6). A paired Student’s t-test confirms an asymmetric distribution of counts of Pax6 expressing cells at these stages, with a higher statistical support and a higher mean AI value at stage 30 than at stage 29 (paired Student’s t-test; n = 3; p-value = 1.257E-02 at stage 29 and 2.315E-02 at stage 30; Fig. 5D; Table S6). In view of this asymmetry, we next focussed on the ratio of BrdU to Pax6 expressing cell count in order to assess proliferation rate asymmetries between the left and right LVZ (Fig. 5F). In all embryos analysed, this ratio appears higher on the right than on the left, with a significant asymmetry in distribution at stage 29 (n = 3; paired Student’s t-test; p-value = 5.859E-03), not observed at stage 30 (Fig. 5F; Table S6). These data highlight a transient asymmetry of the cell proliferation rate, higher in the left LVZ than in the right one at stage 29. In order to assess the contribution of apoptosis to the PNE and LVZ size asymmetries, we also conducted TUNEL assays on habenula sections at stages 27, 29, 30, 31 and 32. Very low numbers of apoptotic cells were detected whether on the right or left side, whatever the territory considered (respectively at most 3, 1 and 4 apoptotic cells per habenula in the PNE, LVZ and other territories; Fig. S2K,L; Table S7), suggesting that apoptosis does not play a major part in the formation of the asymmetries observed.

### Left and right territories of neural progenitors become symmetric following pharmacological treatment with SB-505124

**Figure 6:**
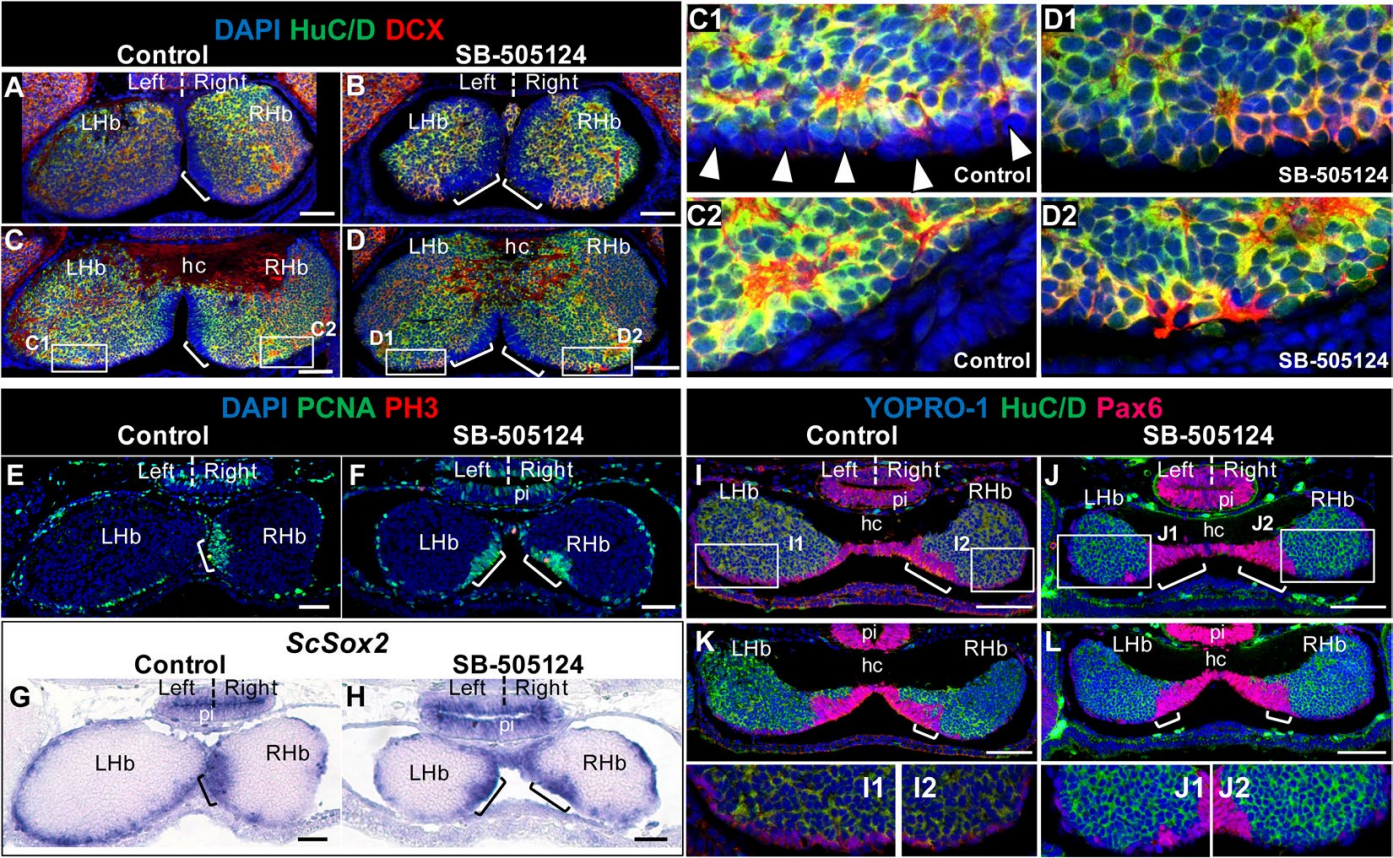
LVZ and PNE neurogenetic asymmetries are dependent on Nodal signalling. Transverse sections of stage 30–31 catshark habenulae following IHC using antibodies against HuC/D and DCX (**A**–**D**), PCNA and PH3 (**E**,**F**), HuC/D and Pax6 (**I**–**L**), or following ISH using a *ScSox2* probe (**G**,**H**). (**A**,**C**,**E**,**G**,**I,K**) and (**B**,**D**,**F**,**H**,**J**,**L**) show sections of control and SB-505124-treated embryos, respectively. (**A**,**B,E**,**F**,**G**,**H,I**,**J**) and (**C,D,K,L**) are respectively located at anterior and medial levels of the habenulae, as defined in Fig. 2G,G1,G2. (**A**–**D**), (**E**–**H**) and (**I**–**L**) respectively correspond to stage 31+, 31 and 30+ embryos. (**C1**,**C2**), (**D1**,**D2**), (**I1**,**I2**) and (**J1**,**J2**) show higher magnifications of LVZ territories boxed in (**C**), (**D**), (**I**) and (**J**) respectively. Symbols and abbreviations are as in Fig. 2. Scale bars = 100 μm.

We previously showed in the catshark that an *in ovo* injection of SB-505124, an antagonist of the TGF-β type I receptors Alk4/5/7^18^, prior to the onset of Nodal activation in the diencephalon, results in a loss of the early left sided Nodal activity, as well as of later habenular asymmetries in size and regional marker expression^5^. In order to test whether the treatment could similarly abolish the neurogenetic asymmetries observed in the PNE and the LVZ, we analysed its effect on the maintenance of these territories at stages 30 to 31. In stage 31 control embryos, HuC/D and DCX immunoreactivity is observed in the right LVZ but largely excluded from the left one (Fig. 6A,C,C1,C2), as observed in untreated embryos. Following SB-505124 treatment, HuC/D and DCX expression appears similar between the left and right LVZ, and comparable to that observed in the right LVZ of control embryos (Fig. 6B,D,D1,D2). *ScSox2* and Pax6 LVZ expression, more patchy on the right than on the left in control embryos (Fig. 6G,I,K,I1,I2), also becomes very similar following SB-505124 treatment (Fig. 6H,J,L,J1,J2). In the PNE, the PCNA positive, HuC/D and DCX negative territory exhibits a marked size asymmetry in control embryos, similar to untreated ones (Fig. 6A,C,E). This asymmetry is lost following SB-505124 treatment, with the left PNE expanding to a size comparable to its counterpart on the right and becoming visible at anterior-most levels (compare Fig. 6B,D,F). Analyses of *ScSox2* and Pax6 PNE expression lead to the same conclusions (compare Fig. 6G–L). These data show that the asymmetric regulation of neural progenitor maintenance in the PNE and LVZ is abolished by the pharmacological treatment. Expression of *Pitx2*, a canonical target of Nodal in left-right asymmetry formation, has been shown to be lost in the catshark following SB-505124 treatment^5^. In order to assess whether this gene may be a candidate to mediate the drug effect, we analysed its expression from stage 27 to 31. *ScPitx2* expression is restricted to the left habenula at all stages analysed (Fig. S3). The signal spans the distal part of the evagination at stage 27 (Fig. S3A,A1). When the LVZ appears, at stage 29, it becomes restricted to the left LVZ and adjacent lateral-most territory of the PNE (Fig. S3B,B1). At stage 31, the signal persists in the left LVZ and lateral PNE (Fig. S3C–E), extending to the whole PNE at medial organ levels (Fig. S3D; compare Fig. S3C–G).

## Discussion

**Figure 7:**
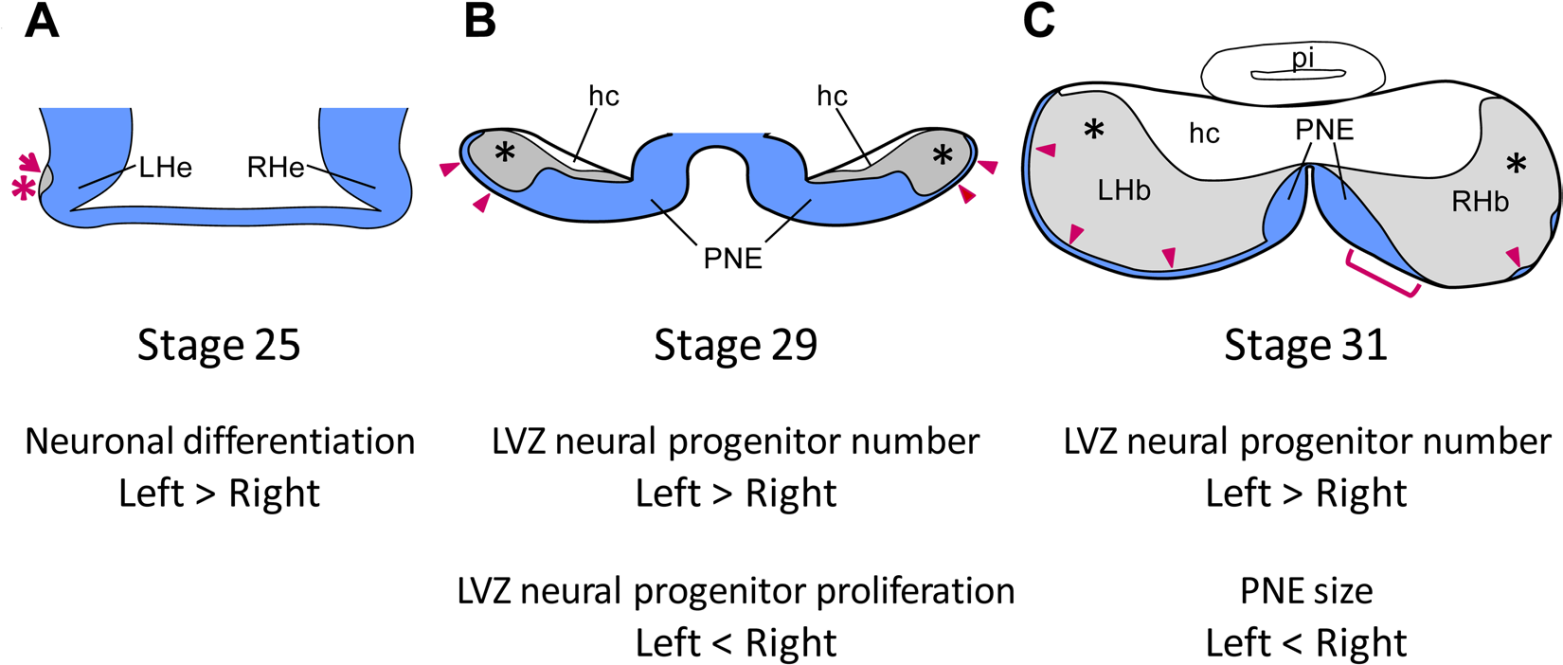
Scheme summarising the neurogenetic asymmetries detected in the catshark developing habenulae. Transverse sections are schematised at stages 25 (**A**), 29 (**B**) and 31 (**C**). Neural progenitor territories are coloured in blue and an asterisk shows territories expressing early neuronal differentiation markers. The red arrow and asterisk in (**A**) indicate a left restricted differentiated territory. Black asterisks in (**B**,**C**) indicate differentiated territories. Red arrowheads in (**B**,**C**) point to LVZ neural progenitors and a red bracket in (**C**) delineates the PNE right size expansion relative to the left.

Our solid understanding of the cellular mechanisms involved in habenular asymmetry formation in the zebrafish contrasts with the paucity of data available in other vertebrates. Our analysis addresses this issue for the first time in a non-teleost model, the catshark, which as a chondrichthyan, provides an excellent reference to infer gnathostome ancestral traits via comparisons with the zebrafish^19^. These data highlight two main neurogenetic asymmetries, which may contribute to the elaboration of habenular asymmetries in the catshark (Fig. 7). We first find a left restricted initiation of neuronal differentiation, observed at stage 25 (Fig. 7A). At later stages, we also detect marked asymmetries in neural progenitor territories, with a higher number of LVZ neural progenitors on the left than on the right starting from stage 29 (Fig. 7B), and a consistent PNE size expansion in the right habenula relative to the left one starting from stage 31 (Fig. 7C). The cellular mechanisms responsible for this differential maintenance between the left and the right habenulae remain to be explored but our analysis provides insight into this question. An asymmetric regulation of apoptosis, known to regulate the number of neural progenitors in the mammalian brain^20,21^ seems unlikely to play a major role, in view of the very small number of apoptotic cells detected at the stages studied. Our analysis also failed to detect significant asymmetries in cell proliferation rate, except for a significantly higher rate observed in the right LVZ at stage 29. This transient cell proliferation asymmetry may contribute to the decrease in magnitude of the LVZ asymmetry with regard to the number of Pax6 positive neural progenitors between stage 29 and 30. However, it cannot explain the preferential maintenance of the LVZ on the left already observed at stage 29 and is difficult to relate to the right PNE extension observed at stage 31. Taken together, these data suggest that another cellular mechanism, such as an asymmetric regulation of cell cycle exits between the left and the right, may be responsible for the differential maintenance of LVZ and PNE neural progenitors. The difference in size of *ScNeuroD1* territory between the left and right habenulae supports this possibility, which will have to be assessed by quantified analyses of the rates of cell cycle exits as neurogenesis proceeds.

We previously observed that *in ovo* injection of the pharmacological inhibitor SB-505124 after neural tube closure, just prior to the onset of a left sided Nodal expression in the diencephalon, results in a loss of habenular asymmetries in size and molecular regionalisation in the catshark, with a right isomerism^5^. Similarly, this pharmacological treatment abolishes the asymmetric maintenance of LVZ and PNE neural progenitors, with a right side phenotype observed in both habenulae. Whether these neurogenetic asymmetries may be dependent on the earlier Nodal diencephalic activity is difficult to formally address in the catshark. However, in support of this hypothesis, the catshark orthologue of *Pitx2*, whose habenular expression is lost following SB-505124 treatment, is selectively expressed in the left LVZ and lateral part of the PNE at the time when neurogenetic asymmetries are detected at this level. This gene, known as a canonical target of Nodal signalling in left-right asymmetry formation, thus appears as a candidate to mediate possible effects of Nodal on the formation of habenular neurogenetic asymmetries in the catshark. An unexpected result of this study is that the injection of the pharmacological inhibitor SB-505124 in the conditions described above has opposite effects on the LVZ and PNE, resulting in an increased cell number in the former, but a smaller size of the latter in the left versus right habenula. Several distinct yet non-mutually exclusive mechanisms could account for these differential effects. First, we cannot formally exclude that the treatment may affect not only the early, left restricted window of Nodal activity immediately following the drug injection, but also other yet unidentified phases of Alk4/5/7 mediated signalling activities, also known to be inhibited by SB-505124^18^, in the right habenula. A bilateral expression of the FoxH1 forkhead activin transducer has thus been observed in the zebrafish^22^. Another possibility may be related to differential sequential effects in the LVZ and the PNE, related to asymmetric modifications of the habenula environment as differentiation proceeds. Temporal regulations of the identity of neural progenitors have indeed been shown to involve dynamic extrinsic signals, such as those secreted by previously generated neurons in a variety of model systems^23^. The asymmetries first observed at stage 25 and detected in the LVZ at stage 29, may similarly modulate those later arising in the PNE in a cell non-autonomous manner. Finally, cell intrinsic genetic programs, possibly differentially interpreting the early phase of Nodal activity, may also differ between the LVZ and PNE. In line with this hypothesis, we observe a prominent left expression of *ScPitx2* in the LVZ, but not in the PNE, prior to stage 31. Further dissections of the mechanisms acting cell autonomously and non-cell autonomously in asymmetry formation in the catshark developing habenulae will be crucial to resolve these issues. Comparisons of the neurogenetic asymmetries identified here in the catshark with the zebrafish also provide insights into the evolution of the mechanisms of habenular asymmetry formation. The early left restricted neuronal differentiation observed in the catshark as in the zebrafish^9,10^, suggests that the asymmetric temporal regulation of neuronal differentiation demonstrated in the latter, may reflect the conservation of an ancestral characteristic of jawed vertebrates. In contrast, neither size asymmetries of neural progenitor territories, nor differences in cell proliferation rate between the left and right developing habenulae as observed in the catshark, were reported in the zebrafish. Whether these traits may correspond to ancestral gnathostome features, or to more recent adaptations of chondrichthyans, remains unknown. Changes in the regulation of neurogenesis have been identified as a driving force of cortical evolution in amniotes^24^ and suggested to contribute to the diversification of habenular asymmetries across vertebrates^1,25^. Our data, which highlight both conserved and divergent neurogenetic asymmetries between the catshark and the zebrafish, support this view. Furthermore, a noticeable feature of the catshark is that the preferential maintenance of proliferating neural progenitors, a process potentially affecting organ size^26^, is not a unilateral one but is observed in the right habenula as well as in the left one, depending on the progenitor pool considered, PNE or LVZ. This provides a rationale to reconcile the apparent discrepancy between the conservation of a left restricted Nodal activity and variations of the laterality of size asymmetries, as observed between the lamprey and catshark^1,5,25^. Evolutionary variations affecting the size ratio between two such progenitor pools, or their temporal regulation, could indeed result in changes in the laterality of habenular size asymmetries across vertebrates. Finally, an important remaining question concerns the contribution of the neurogenetic asymmetries characterised in the catshark to the differences in molecular organisation between the left and right habenulae. The coupling of marked neurogenetic asymmetries, as observed here, and of a temporal regulation of cell fate choices, as frequently encountered in the central nervous system^27^ and as reported in the zebrafish developing habenulae^9,10^, may be the major mechanism controlling habenular asymmetry formation in the catshark. However, we cannot exclude that an additional asymmetric regulation of cell fate choices, involving a left repression of Wnt signalling as reported in the zebrafish^8^, may contribute to this process, possibly under the control of Nodal rather than a parapineal secreted signal. Direct perturbations of neurogenesis, for instance by manipulations of Notch activity as described in the zebrafish^9^, and analyses of the regulation of cell fate choices will be crucial to resolve this issue.

## Methods

### Embryo collection

*Scyliorhinus canicula* eggs were obtained from Roscoff and Banyuls sur Mer Marine Stations. Embryos were dissected, fixed and staged as described previously^5^.

### *In situ* hybridization (ISH)

ISH of sections were conducted using digoxigenin-labelled antisense RNAs as previously described^28,29^. Probes were obtained from collections of embryonic *S. canicula* cDNA recombinants, characterized by Sanger sequencing and described in^30^. Clone identities were confirmed by phylogenetic analyses (Fig. S4). Accession numbers for the sequences used in this study are listed in Table S10. Following ISH, nuclei were counterstained using Nuclear Fast Red (Sigma N3020). For all probes analysed, the orthogonality of section planes to the antero-posterior axis (as depicted in Figs 1 and 2) was checked using symmetrical morphological references (eyes, olfactory epithelium) and sections were systematically analysed at all organ levels (at least one section every three successive sections at stages 27–28 and one every four sections at stages 29–31; see example in Fig. S5).

### Immunohistochemistry (IHC)

The antibodies used and their concentration are listed in Supplementary Information (Table S9). Fluorescent immunohistochemical analyses of sections were conducted as previously described^5^ for PCNA, PH3, Pax6, HuC/D and DCX IHC. BrdU pulse labelling were conducted by incubating catshark embryos in 10 mg/ml BrdU in oxygenated artificial sea water after opening the egg shell. Incubations were conducted for 30, 45, 120 and 150 minutes respectively at stages 27, 28, 29 and 30 respectively. For detection of BrdU, sections were incubated in 2 N HCl for 30 minutes at 50 °C prior to fluorescent IHC analysis in the conditions described above. Embryo sections were imaged with a Leica SP8 confocal laser-scanning microscope and images processed using ImageJ. As for ISH, at least one section every three (stages 27–28) or four (stages 29–31) successive sections was analysed, over the whole organ.

### TUNEL assays

Apoptosis was detected on paraffin-embedded sections with the *In Situ* Cell Death Detection Kit (Roche, 11684809910) according to the supplier’s instructions. In short, slides were pretreated in 0.1 M citrate buffer pH6.0 with a 350 W microwave irradiation for 5 minutes, incubated in the TUNEL reaction mixture for 1 h at 37 °C prior to DAPI nuclear staining. For positive controls, a DNase I treatment (1000 U/mL) was added for 10 min at room temperature prior to the labeling procedure. For negative controls, slides were treated as described above, except that terminal transferase was omitted in the reaction.

### Countings and data analysis

Following fluorescent IHC and DAPI staining, PH3 positive nuclei in the PNE and LVZ were counted manually on adjacent habenular transverse sections using an epifluorescence Olympus IX51. Countings were restricted to the habenular evaginations, taking into account all sections (10 µm thick) starting from the anterior part of the organ (total number of sections = 25 at stage 27, 32 at stage 29, 35 at stage 30, 40 at stage 31). The same methodology was used to estimate the number of PH3 positive nuclei in the right PNE expansion, defined by comparisons between the left and the right habenulae on sections submitted to fluorescent IHC using antibodies directed against PCNA and PH3. For statistical analyses, these counts were systematically corrected and divided by a factor of 2, in order to take into account the relative thickness of nuclei and sections according to^31^. BrdU and Pax6 positive cells were counted in the whole habenular evaginations as described above, except that only one section was taken into account every three (stage 28) or four (stages 29–30) successive sections. No correction for section thickness was applied in this case before statistical analysis. Ratios of BrdU to Pax6 expressing cell counts were calculated using counts in the whole habenula, obtained as described above. X^2^ statistical tests were conducted for individual embryos using the count data obtained as described above, following correction in the case of PH3 labelling, the null hypothesis for each embryo analysed being that cell counts are identical in the right and in the left habenulae. For Student’s t-tests, counts obtained for replicates of the same condition (same zone, same stage, same side) were taken into account in order to assess the probability of occurrence of the observed distribution against a symmetry null hypothesis (same mean number of cell counts or ratios on the left and on the right for the group of embryos considered). All statistical tests were performed using the R statistical software^32^ with the following parameters (α = 5%; for X^2^ test df = 1).

### Pharmacological treatments

Catshark embryos were treated by *in ovo* injection of SB-505124, a selective inhibitor of TGF-β type I receptors Alk4/5/7^18^, as previously described^5^. The same protocol was applied to control embryos, except for the absence of the drug in the injected solution.

### Data availability statement

The data, probes and protocols used in this study are available upon request to SM.

## Electronic supplementary material

Supplementary Information
