# Supplementary material for: Neurogenetic asymmetries in the catshark developing habenulae: mechanistic and evolutionary implications
Source: Sci Rep. 2018 Mar 15;8:4616. doi: 10.1038/s41598-018-22851-3 (PMC5854604; doi:10.1038/s41598-018-22851-3)
Supplement: Supplementary file 1 — Supplementary Information [file 41598_2018_22851_MOESM1_ESM.pdf]

Neurogenetic asymmetries in the catshark developing habenulae: mechanistic and evolutionary implications

Ronan Lagadec, Maxence Lanoizelet, Nuria Sánchez-Farías, Fanny Hérard, Arnaud Menuet, Hélène Mayeur, Bernard Billoud, Isabel Rodriguez-Moldes, Eva Candal and Sylvie Mazan

LEGENDS TO SUPPLEMENTARY FIGURES

Fig. S1. Histological structure of developing habenulae in the catshark *S. canicula*

Transverse sections through the catshark habenulae at stages 27 (A), 28 (B), 29 (C), 30 (D) and 31 (F,G) following hematoxylin staining (12 µm sections). (E) Scheme showing a lateral view of the developing catshark brain at stages 31 indicating the plane, level and tissue organisation (boxed) of the sections shown in (F-G). (C1) is a higher magnification of a stage 29 left habenula (5 µm section), showing the difference in cell organisation between the PNE and lateral territory of dispersed rounded cells. A thin layer of distinct ventricular cells (LVZ) extends laterally to the PNE, adjacent to rounded cells (see Figure 2). (F1) and (G2) show higher magnifications of the PNE territories boxed in (F) and (G), respectively. (G1) is a higher magnification of the lateral territory boxed in (G). This territory is labelled by asterisks in (A-D,F,G). Black arrowheads in (A-D,F,G) point to the boundary between the habenulae and the forming choroid plexus. Abbreviations used: Cp, choroid plexus; hc, habenular commissure; LVZ, lateral ventricular zone; LHb, left habenula; RHb, right habenula; pi, pineal stalk; PNE, pseudo-stratified neuroepithelium. Scale bars=100µm.

Fig. S2. Expression of neurogenetic genes and proliferation-differentiation markers in the developing catshark habenulae

Transverse sections through the catshark habenulae following ISH with probes for *ScNgn2* (A-E) and *ScNeuroD1* (F-J), a TUNEL assay for apoptosis detection (K-L) or IHC using antibodies directed against PCNA and PH3 (M; PCNA in green and PH3 in red). (K-M) nuclei were counterstained with DAPI (blue). Stages are; 25 (A,F), 26 (B,G), 27 (C,H), 29 (D,I,K), 31 (E,J,L) and 32 (M). Section planes in (E,J,L-M) are the same as in Fig. S1. (M), (E,J) and (L) correspond to anterior, medial and posterior levels of the organ, respectively. The bracket in (E,K,L) delineates an extension of the PNE in the right but not the left habenula. Black arrows in (J) delimit the boundaries of *ScNeuroD1* expression territory outside the PNE: the labelled territory is broader on the left than on the right at stage 31. This difference is already observed at stage 30 (not shown). White arrows in (K,L) point cells positive for the TUNEL assay. Symbols and abbreviations are the same as in Fig. S1. Scale bars=100µm.

Fig. S3. Comparison of *ScPitx2* and *ScPNCA* expressions in the catshark developing habenulae

Transverse sections of catshark habenulae following ISH with a *ScPitx2* probe at stages 27 (A), 29 (B) and 31 (C-E). (F-H) transverse sections of the same embryo, adjacent to those shown in (C-E), following ISH with a *ScPCNA* probe. (C,F), (D,G) and (E-H) correspond to sections at anterior, medial and posterior levels of the organ, respectively. (A1,A2) and (B1,B2) show higher magnifications of the territories boxed in (A) and (B), respectively. The bracket in (F-H) shows the lateral extension of the PNE observed in the right habenula but not the left. *ScPCNA* and *ScPitx2* territories overlap on the left at lateral levels of the PNE. *ScPitx2* also shows strong expression in the left LVZ. These *ScPitx2*

territories exhibit neurogenetic asymmetries at stage 31. Abbreviations are the same as in Fig. S1. Scale bars=100μm.

<u>Fig. S4</u>. Phylogenetic analyses

Maximum-likelihood phylogenetic trees for Neurogenin (A), Sox1/2/3 (B), NeuroD (C) and PCNA (D). Amino acid sequences of gene family members were extracted from Genbank or Ensembl protein databases, or inferred from cDNA sequences. Alignments were obtained using the MUSCLE package [Elgar 2004] and BioEdit [Tippmann 2004] was used for manual corrections. Trees were constructed using PhyML (version 3.0), integrated into Seaview 4.2, using the Maximum Likelihood method and the LG-F+Γ_{12}+I substitution model. SPR was used to compute the trees. The trees were viewed and edited with the TreeExplorer program in MEGA 6.0 [Kumar et al. 2008]. Posterior probabilities for each node were calculated *a posteriori* and are displayed (in %) when higher than 80%. Accession numbers and nomenclature for the sequences used in the phylogenetic reconstructions are listed in Table S10. Abbreviations used: Ac, *Anolis carolinensis*; Bf, *Branchiostoma floridae*; Cm, *Callorhinchus milii*; Dr, *Danio rerio*; Gg, *Gallus gallus*; Hs, *Homo sapiens*; Lc, *Latimeria chalumnae*; Le, *Leucoraja erinacea*; Lo, *Lepisosteus oculatus*; Ol, *Oryzias latipes*; Pm, *Petromyzon marinus*; Ps, *Pelodiscus sinensis*; Sc, *Scyliorhinus canicula*; Xt, *Xenopus tropicalis*.

<u>Fig. S5.</u> Transverse sections of stage 31 catshark habenulae following ISH with a *ScSox2* probe.

(A-B) and (C-V) show transverse sections of the head and of the habenulae respectively (section plane shown in Fig. S1). (C) to (V) correspond to different levels of the organ, from anterior to posterior. (D) and (O) are magnifications of the habenula territory boxed in (A) and (B). Only one every two sections was submitted to ISH and the *ScSox2* profiles thus obtained are shown for all sections analysed. A bracket delineates the expansion displayed by the right habenula compared to the left one. Schemes describing the morphological of habenulas at this stage are presented in Fig. S1. Abbreviations: same as in Fig.S1; Mes, mesencephalon; OB, olfactory bulb; OE, olfactory epithelium. Scale bars=100μm.

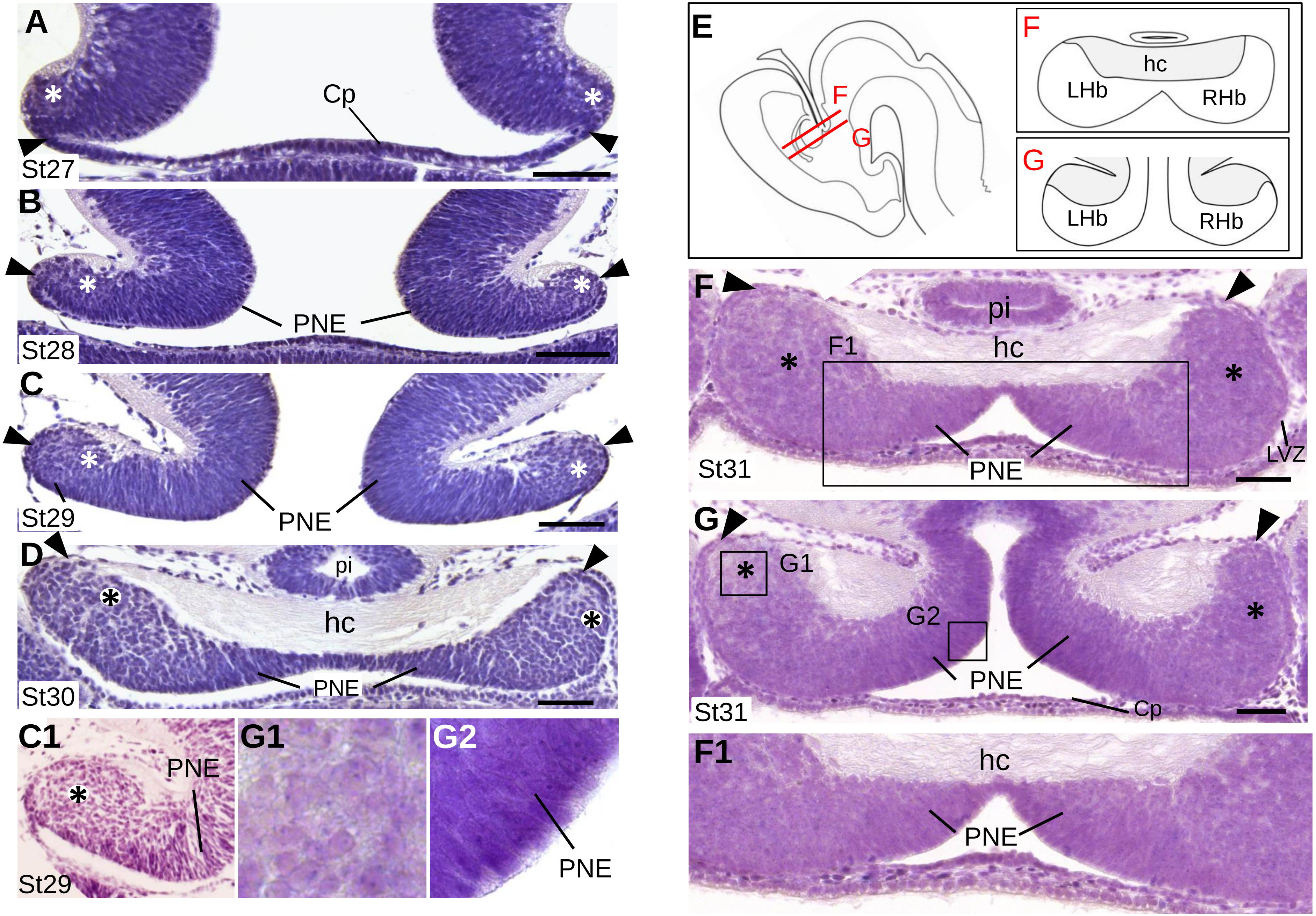
A
Cp
St27
B
PNE
St28
C
PNE
St29
D
pi
hc
PNE
St30
C1
PNE
St29
G1
G2
PNE
E
F
G
hc
LHb
RHb
F
pi
F1
hc
PNE
LVZ
St31
G
G1
G2
PNE
Cp
St31
F1
hc
PNE

Figure S1

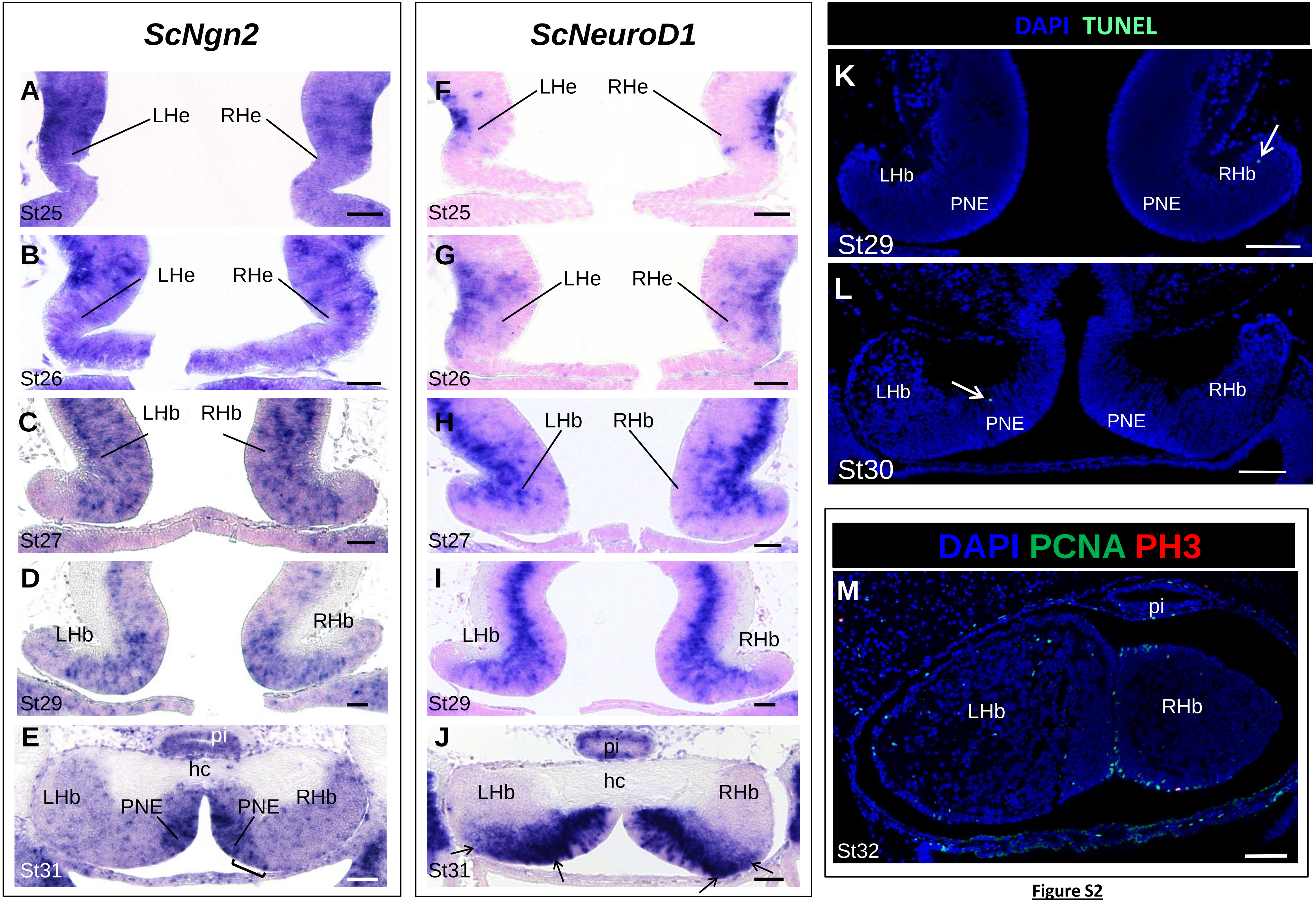

ScNgn2
A
LHe
RHe
St25
B
LHe
RHe
St26
C
LHb
RHb
St27
D
LHb
RHb
St29
E
pi
hc
LHb
PNE
PNE
RHb
St31
ScNeuroD1
F
LHe
RHe
St25
G
LHe
RHe
St26
H
LHb
RHb
St27
I
LHb
RHb
St29
J
pi
hc
LHb
RHb
St31
DAPI TUNEL
K
LHb
PNE
PNE
RHb
St29
L
LHb
PNE
PNE
RHb
St30
DAPI PCNA PH3
M
pi
LHb
RHb
St32

Figure S2

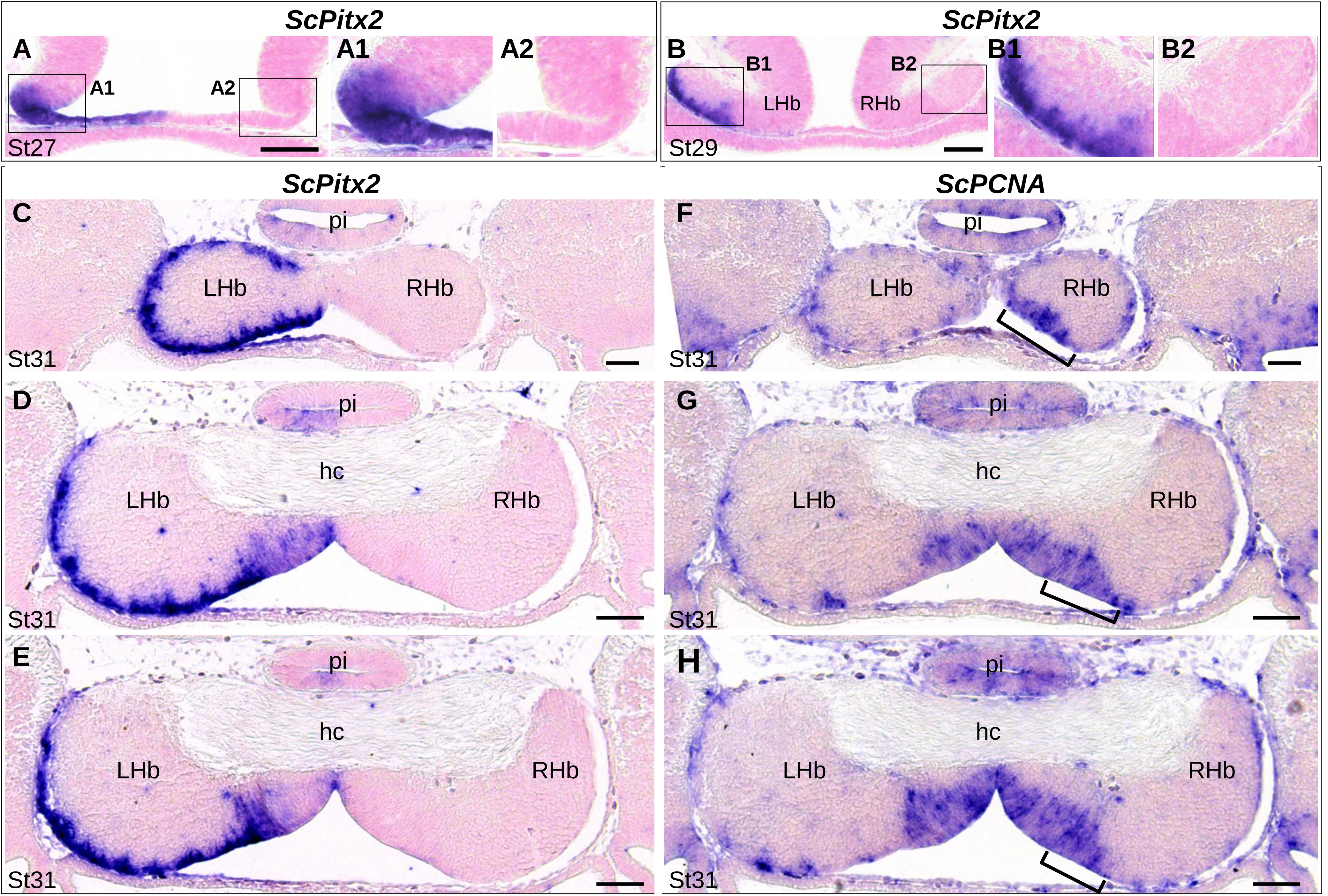
ScPitx2
A
A1
A2
St27
A1
A2
ScPitx2
B
B1
B2
LHb
RHb
St29
B1
B2
ScPitx2
C
pi
LHb
RHb
St31
D
pi
hc
LHb
RHb
St31
E
pi
hc
LHb
RHb
St31
ScPCNA
F
pi
LHb
RHb
St31
G
pi
hc
LHb
RHb
St31
H
pi
hc
LHb
RHb
St31

Figure S3

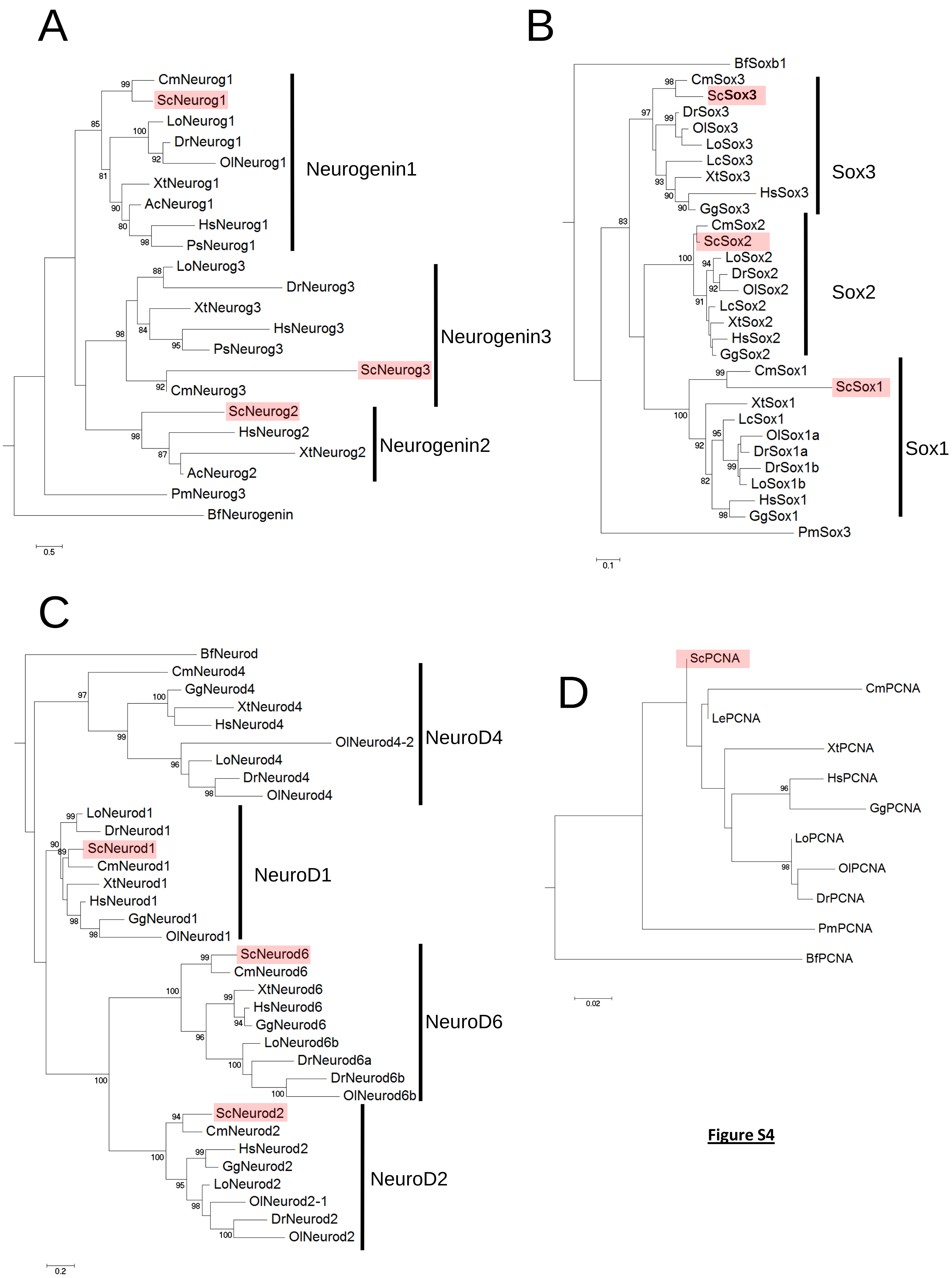
A
CmNeurog1
ScNeurog1
LoNeurog1
DrNeurog1
OlNeurog1
XtNeurog1
AcNeurog1
HsNeurog1
PsNeurog1
Neurogenin1
LoNeurog3
DrNeurog3
XtNeurog3
HsNeurog3
PsNeurog3
ScNeurog3
CmNeurog3
Neurogenin3
ScNeurog2
HsNeurog2
XtNeurog2
AcNeurog2
Neurogenin2
PmNeurog3
BfNeurogenin
0.5
B
BfSoxb1
CmSox3
ScSox3
DrSox3
OlSox3
LoSox3
LcSox3
XtSox3
HsSox3
GgSox3
Sox3
CmSox2
ScSox2
LoSox2
DrSox2
OlSox2
LcSox2
XtSox2
HsSox2
GgSox2
Sox2
CmSox1
ScSox1
XtSox1
LcSox1
OlSox1a
DrSox1a
DrSox1b
LoSox1b
HsSox1
GgSox1
Sox1
PmSox3
0.1
C
BfNeurod
CmNeurod4
GgNeurod4
XtNeurod4
HsNeurod4
OlNeurod4-2
LoNeurod4
DrNeurod4
OlNeurod4
NeuroD4
LoNeurod1
DrNeurod1
ScNeurod1
CmNeurod1
XtNeurod1
HsNeurod1
GgNeurod1
OlNeurod1
NeuroD1
ScNeurod6
CmNeurod6
XtNeurod6
HsNeurod6
GgNeurod6
LoNeurod6b
DrNeurod6a
DrNeurod6b
OlNeurod6b
NeuroD6
ScNeurod2
CmNeurod2
HsNeurod2
GgNeurod2
LoNeurod2
OlNeurod2-1
DrNeurod2
OlNeurod2
NeuroD2
0.2
D
ScPCNA
CmPCNA
LePCNA
XtPCNA
HsPCNA
GgPCNA
LoPCNA
OlPCNA
DrPCNA
PmPCNA
BfPCNA
0.02

Figure S4

ScSox2, stage 31

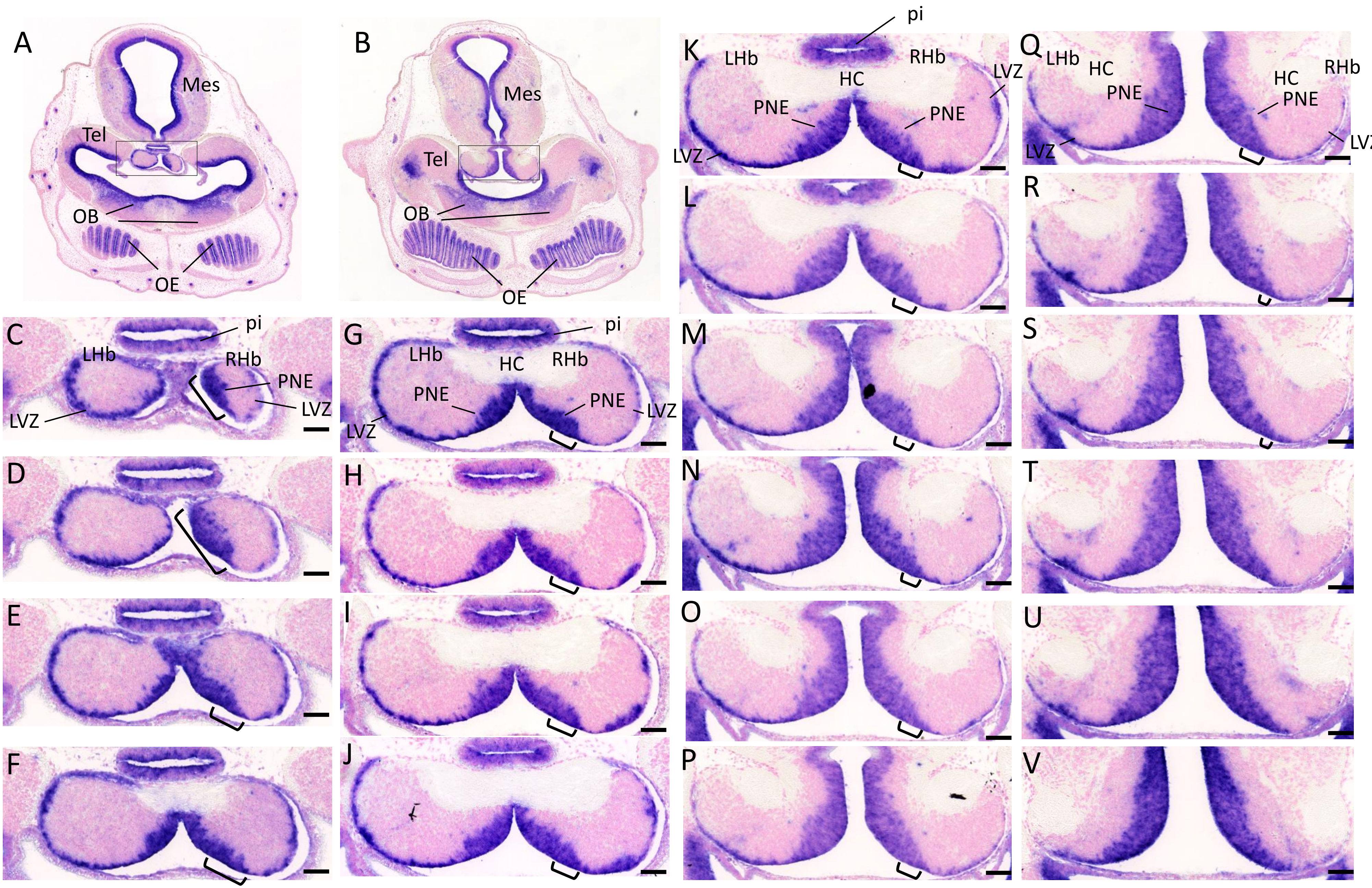

Figure S5

SUPPLEMENTARY TABLES

Stage 27				Stage 28				Stage 29			
**Embryo**	**Left**	**Right**	**p-value**	**Embryo**	**Left**	**Right**	**p-value**	**Embryo**	**Left**	**Right**	**p-value**
n°27.6	60	61	0.93	n°28.1	51	47	0.69	n°29.9	50	53	0.77
n°27.7	59	63	0.72	n°28.2	47	48	0.92	n°29.10	46	54	0.42
n°27.8	64	57	0.52	n°28.3	46	48	0.84	n°29.11	49	50	0.92

	Stage 30				Stage 31		
**Embryo**	**Left**	**Right**	**p-value**	**Embryo**	**Left**	**Right**	**p-value**
n°30.7	53	56	0.77	n°31.11	60	62	0.86
n°30.8	56	55	0.92	n°31.12	61	64	0.79
n°30.9	54	53	0.92	n°31.13	67	63	0.73

Table S1. Estimation of cell densities in the left and right developing habenulae

Quantifications were performed using images of habenula sections following DAPI or YOPRO-1 nuclear staining. For each embryo analysed (n=3 at each stage), a 10µm section was selected at a medial level of the developing habenulae and the total number of nuclei contained within a squared area of the PNE (50µm x 50µm) was counted on the left and on the right. These counts are indicated in the table, for each embryo analysed. X^2 tests, performed on cell counts for each individual embryo, showed no difference between the left and the right sides (p-values comprised between 0.4 and 1.0).

**Stage 27**								
	**PNE**				**LVZ**			
	**Left**	**Right**	**AI (%)**	**X² p-value**	**Left**	**Right**	**AI (%)**	**X² p-value**
n°27.1	235	218	3.8	0.5722	-	-	-	-
n°27.2	193	194	-0.3	0.9713	-	-	-	-
n°27.3	107	117	-4.5	0.6366	-	-	-	-
n°27.4	107	99	3.9	0.6935	-	-	-	-
n°27.5	246	252	-1.2	0.8492	-	-	-	-
n°27.6	190	183	1.9	0.7977	-	-	-	-
n°27.7	212	209	0.7	0.9177	-	-	-	-
n°27.8	142	146	-1.4	0.8676	-	-	-	-
**Stage 29**								
	**PNE**				**LVZ**			
	**Left**	**Right**	**AI (%)**	**X² p-value**	**Left**	**Right**	**AI (%)**	**X² p-value**
n°29.1	229	143	23.1	1.616E-03	14	16	-6.7	0.7963
n°29.2	389	273	17.5	1.433E-03	30	16	30.4	0.1444
n°29.3	297	303	-1.0	0.3768	29	16	28.9	0.1706
n°29.4	320	316	0.6	0.9107	24	17	17.1	0.4395
n°29.5	230	238	-1.7	0.7937	21	15	16.7	0.4795
n°29.6	221	215	1.4	0.839	14	12	7.7	0.7815
n°29.7	289	266	4.1	0.49	24	20	9.1	0.6698
n°29.8	285	270	2.7	0.6525	19	15	11.8	0.6276
**Stage 30**								
	**PNE**				**LVZ**			
	**Left**	**Right**	**AI (%)**	**X² p-value**	**Left**	**Right**	**AI (%)**	**X² p-value**
n°30.1	180	171	2.6	0.7341	13	9	18.2	0.5465
n°30.2	293	311	-3.0	0.6045	24	25	-2.0	0.9195
n°30.3	181	187	-1.6	0.825	14	15	-3.4	0.8955
n°30.4	151	155	-1.3	0.8715	19	16	8.6	0.7199
n°30.5	88	91	-1.7	0.874	15	16	-3.2	0.8989
n°30.6	199	207	-2.0	0.7789	15	14	3.4	0.8955
**Stage 31**								
	**PNE**				**LVZ**			
	**Left**	**Right**	**AI (%)**	**X² p-value**	**Left**	**Right**	**AI (%)**	**X² p-value**
n°31.1	127	267	-35.5	6.123E-07	33	19	26.9	0.1698
n°31.2	72	103	-18.7	9.752E-02	33	8	61.0	5.766E-03
n°31.3	162	223	-15.8	2.793E-02	41	26	22.4	0.195
n°31.4	108	163	-20.3	1.815E-02	18	10	28.6	0.285
n°31.5	72	113	-22.2	3.305€-03	21	9	40.0	0.1213
n°31.6	84	148	-27.6	2.967E-03	25	12	35.1	0.1307
n°31.7	81	136	-25.3	8.289E-03	28	16	27.3	0.2008
n°31.8	79	115	-18.6	6.761E-02	13	9	18.2	0.5465
n°31.9	52	85	-24.1	4.62E-02	14	4	55.6	9.558E-02
n°31.10	141	178	-11.6	0.143	29	15	31.2	0.1356

Table S2. Counts of PH3 positive nuclei and asymmetry index in the developing habenulae of stage 27 to 31 catshark embryos

For each individual embryo (1st column), PH3 positive nuclei were counted in the PNE at stages 27, 29, 30 and 31 (n=8,8,6 and 10 respectively), and in the LVZ at stages 29, 30 and 31 (n=8,6 and 10 respectively) from serial 10µm habenular sections. All habenular sections were taken into account in these counts. For each embryo analysed, the degree of asymmetry with regard to the number of PH3 positive cells in the PNE and in the LVZ was quantitatively expressed as an asymmetry index (AI), defined as the difference between counts of PH3 positive cells on the left versus the right side, normalised to the total number of PH3 positive cells. AI values in % are shown in the 4th column (PNE) and the 8th column (LVZ), respectively. Following count correction to take section thickness into account, X^2 test p-values were calculated for each embryo in order to detect statistically significant count differences between the left and the right (threshold =5E-02, green shading).

	Student's t-test (count data)	
	PNE	LVZ
stage 27	5,9050E-01	-
stage 29	1,1240E-01	1,5950E-02
stage 30	2,1900E-01	4,0170E-01
stage 31	4,0810E-04	4,1650E-05

<u>Table S3.</u> Statistical analysis of habenular asymmetry with regard to PH3 cell counts in the PNE and LVZ of stage 27 to 31 catshark embryos

The table shows the results of statistical tests of habenular asymmetry in the PNE (stages 27 to 31; column 2) and in the LVZ (stage 29 to 31; column 3), with regard to corrected PH3 cell counts of Table S2. For each condition (same zone and same stage), a paired Student's t-test was conducted in order to test whether the distribution of PH3 positive nuclei counts significantly differed between the left and the right. The corresponding p-values are shown in columns 3 and 6. P-values lower than 5E-02 were considered as indicative of a significant asymmetry and are shaded in green.

Stage 28				Stage 29				Stage 30			
**Embryo n°**	**Left**	**Right**	**AI (%)**	**Embryo n°**	**Left**	**Right**	**AI (%)**	**Embryo n°**	**Left**	**Right**	**AI (%)**
n°28.1	507	502	0.5	n°29.9	954	931	1.2	n°30.7	898	896	0.1
n°28.2	563	562	0.1	n°29.10	1171	1161	0.4	n°30.8	1018	1032	-0.7
n°28.3	629	659	-2.3	n°29.11	736	704	2.2	n°30.9	917	887	1.7

<u>Table S4.</u> Counts of BrdU labeled nuclei and asymmetry index in the PNE of catshark developing habenulae at stage 28-30

For each individual embryo (1st, 5th and 9th columns respectively at stages 28, 29 and 30), BrdU positive nuclei were counted and summed in the PNE following IHC with an antibody directed against BrdU. For each embryo analysed, the degree of asymmetry was quantitatively expressed as an asymmetry index (AI), defined as the difference between the number of BrdU positive cells on the left (2nd, 6th and 10th columns) versus the right (3rd, 7th and 11th columns) side, normalised to the total number of BrdU positive cells. AI values in % are shown in the 4th (stage 28), 8th (stage 29) and 12th (stage 30) columns. All X^2 tests, performed on individual embryos, showed an absence of significant difference in the number of labelled nuclei between the left and the right (p-values comprised between 0.399 and 0.976).

PNE, stage 31					
	Left, total	Right, total	Right, expansion	Right, total - Right, expansion	X² p-value right (total - expansion) vs left (total)
n°31.8	79	115	37	78	0.955
n°31.9	52	85	29	56	0.7855
n°31.10	141	178	36	142	0.9665

Table S5. Contribution of the right PNE expansion to the counts of PH3 positive cells in the right PNE

For each of the three stage 31 embryos shown, (1st column), PH3 positive nuclei were counted in the left and right PNE (2nd and 3rd columns). The expansion of the right PNE relative to the left was delineated based on PCNA immunoreactivity and PH3 positive nuclei were counted in this domain (4th column). The counts shown in the 5th column show the difference between the total count of PH3 cells in the right PNE and the latter value. X^2 tests, performed for each individual embryo on cell counts in the left PNE (2nd column) and the corresponding territory on the right (5th column), showed no statistically significant difference, supporting the conclusion that the higher number of PH3 positive cells in the right versus left PNE is due to the larger size of the former.

Stage 29								
	Left			Right			Pax6 AI (%)	X² p-value Pax6
	BrdU	Pax6	BrdU/Pax6 ratio (%)	BrdU	Pax6	BrdU/Pax6 ratio (%)		
n°29.9	74	380	19.5	52	210	24.8	28.8	2.582E-12
n°29.10	80	352	22.7	65	225	28.9	22.0	1.243E-07
n°29.11	81	481	16.8	63	292	21.6	24.5	1.062E-11
**Stage 30**								
	Left			Right			Pax6 AI (%)	X² p-value Pax6
	BrdU	Pax6	BrdU/Pax6 ratio (%)	BrdU	Pax6	BrdU/Pax6 ratio (%)		
n°30.7	80	498	16.1	47	362	12.9	15.8	3.525E-06
n°30.8	89	345	25.8	67	256	26.2	14.8	2.83E-04
n°30.9	71	496	14.3	57	341	16.7	18.5	8.434E-08

Table S6. Counts of BrdU and Pax6 labeled nuclei in the LVZ of catshark developing habenulae at stages 29-30

For each individual embryo (1st column), nuclei positive for the presence of BrdU (left, 2nd column; right, 5th column) and Pax6 (left, 3rd column; right, 6th column) were counted in the LVZ at stages 29 and 30 following IHC with antibodies respectively directed against BrdU and Pax6. The corresponding ratio (percentage of BrdU positive cells to Pax6 expressing ones) is shown in 4th (left) and 7th (right) columns. The degree of asymmetry with respect to Pax6 positive cell counts is quantitatively expressed as the asymmetry index (AI) shown in the 8th column, defined as the difference between the number of positive cells on the left versus the right side, normalised to the total number of positive cells. X^2 tests, performed for each individual embryo on Pax6 positive cell counts (9th column) show a statistically significant difference in Pax6 positive cell counts between the left and the right LVZ in all embryos analysed at stage 29 and 30. An asymmetry in the distribution of Pax6 positive cell counts is also supported by paired Student's t-tests at stage 29 (p-value=0.01257) and at stage 30 (p-value=0.02315). Paired Student's t-tests show a statistically significant asymmetry in the distribution of BrdU/Pax6 ratios at stage 29 (p-value=0.005869) but not at stage 30 (p-value=0.9551).

	**PNE**		**LVZ**		**Other**		**Total**	
	**Left**	**Right**	**Left**	**Right**	**Left**	**Right**	**Left**	**Right**
Stage 27	0	0	0	0	0	0	0	0
Stage 29	0	3	1	0	1	1	2	5
Stage 30	1	0	1	0	4	4	6	4
Stage 31	1	1	0	1	3	2	4	4
Stage 32	0	0	0	0	4	3	4	3

Table S7. Counts of apoptotic cells in the developing habenulae of stage 27 to 31 catshark embryos

For each stage (1st column), apoptotic cells were counted in the PNE (2nd and 3rd columns), LVZ (4th and 5th columns) and in the other territories (6th and 7th columns) of the left (2nd, 4th and 6th columns) and right (3rd,5th and 7th columns) habenulae of a single embryo following a TUNEL assay. For each stage, the total number of apoptotic cells in the left and right habenulae is shown in the 8th and 9th columns respectively. The TUNEL assay was done on 10µm habenular sections. At each stage, all habenular sections were taken account.

	PNE St. 31			LVZ St. 30 and St. 31		
	Untreated	Control	SB-505124	Untreated	Control	SB-505124
	Asymmetric phenotype	Asymmetric phenotype	Right isomerism	Asymmetric phenotype	Asymmetric phenotype	Right isomerism
ScSox2	1/1	3/3	2/2	4/4	3/3	2/2
ScNgn2	1/1	1/1	1/1	-	-	-
ScNeuroD1	1/1	2/2	1/1	-	-	-
ScPCNA	1/1	-	-	-	-	-
**PCNA**	9/9	1/1	1/1	-	-	-
**HuC/D**	2/2	1/1	1/1	2/2	1/1	1/1
**DCX**	1/1	1/1	1/1	4/4	1/1	1/1
**Pax6**	3/3	1/1	1/1	3/3	1/1	1/1
**Embryos analysed, total**	17/17	7/7	6/6	11/11	5/5	4/4

Table S8. Number of embryos analysed and habenular phenotypes observed in the PNE and LVZ
For each marker tested (1st column), ratios show the number of embryos exhibiting the phenotypes indicated in the 3rd line relative to the total number of embryos analysed. These ratios are shown for phenotypes observed in the PNE at stage 31 (2nd, 3rd and 4th columns) and in the LVZ at stages 30-31 (5th, 6th and 7th columns), in three conditions: untreated embryos (2nd and 5th columns), control, DMSO-injected embryos (3rd and 6th columns) and SB-505124 treated embryos (4th and 7th columns). The asymmetric phenotype in the PNE refers to a right expansion relative to the left, as described in Fig. 3, Fig. S2 and S5. The asymmetric phenotype in the LVZ refers to the preferential maintenance of neural progenitors on the left relative to the right as described in Results and Fig. 2.

**Anti-acetylated tubulin**	**Sigma T-6793**	**1:1000**
**Anti-proliferating cell nuclear antigen (PCNA)**	**Sigma P8825**	**1:300**
**Anti-Histone H3 (phospho-S10)**	**Abcam ab5176**	**1:400**
**Anti-human HuC/D**	**Molecular Probes A-21271**	**1:200**
**Anti-doublecortin (DCX)**	**Santa Cruz Biotechnology**	**1:300**
**Anti-Pax6**	**Abcam ab6326**	**1:200**
**Anti-BrdU**	**Abcam ab6326**	**1:100**

Table S9. Antibodies used
For each antibody (1st column), the reference and concentration used are shown in 2nd and 3rd columns respectively.

NEUROGENIN		NEUROD		SOX1/2/3	
CmNeurog1	XM_007901395.1	BfNeurod	XM_002586803.1	BfSoxb1	DQ644541.1
ScNeurog1	KX592193	CmNeurod4	XM_007886134.1	CmSox3	XM_007892868.1
LoNeurog1	ENSLOCT00000014228	GgNeurod4	ENSGALT00000041276	ScSox3	KX592197
DrNeurog1	ENSDART00000078563	XtNeurod4	ENSXETT00000014760	DrSox3	ENSDART00000075617
OlNeurog1	ENSORLT00000025439	HsNeurod4	ENSG00000123307	OlSox3	ENSORLT00000002213
XtNeurog1	ENSXETT00000017331	OlNeurod4-2	ENSORLT00000024677	LoSox3	ENSLOCT00000021635
AcNeurog1	ENSACAT00000001181	LoNeurod4	ENSLOCT00000022352	LcSox3	ENSLACT00000007556
HsNeurog1	ENST00000314744	DrNeurod4	ENSDART00000145072	XtSox3	ENSXETT00000016558
PsNeurog1	ENSPSIT00000004407	OlNeurod4	ENSORLT00000009362	HsSox3	ENST00000370536
LoNeurog3	ENSLOCT00000014195	LoNeurod1	ENSLOCT00000021971	GgSox3	AB011803.1
DrNeurog3	ENSDART00000002863	DrNeurod1	ENSDART00000011837	CmSox2	XM_007907767.1
XtNeurog3	ENSXETT00000055709	ScNeurod1	KX592190	ScSox2	KX592196
HsNeurog3	ENST00000242462	CmNeurod1	XM_007884678.1	LoSox2	ENSLOCT00000021488
PsNeurog3	ENSPSIT00000001125	XtNeurod1	ENSXETT00000030713	DrSox2	ENSDART00000104493
ScNeurog3	KX618851	HsNeurod1	ENST00000295108	OlSox2	ENSORLT00000014648
CmNeurog3	XM_007897988.1	GgNeurod1	ENSGALT00000014484	LcSox2	ENSLACT00000007461
ScNeurog2	KX592194	OlNeurod1	ENSORLT00000013242	XtSox2	ENSXETT00000004031
HsNeurog2	ENST00000313341	ScNeurod6	KX592192	HsSox2	ENST00000431565
XtNeurog2	ENSXETT00000052002	CmNeurod6	XM_007900862.1	GgSox2	ENSGALT00000014379
AcNeurog2	ENSACAT00000026370	XtNeurod6	ENSXETT00000050601	CmSox1	XM_007891764.1
PmNeurog3	ENSPMAT00000006843	HsNeurod6	ENST00000297142	ScSox1	KX618852
BfNeurogenin	XM_002596198.1	GgNeurod6	ENSGALT00000019978	XtSox1	ENSXETT00000004030
		LoNeurod6b	ENSLOCT00000015959	LcSox1	ENSLACT00000006057
**PCNA**		DrNeurod6a	ENSDART00000098859	OlSox1a	ENSORLT00000015157
ScPCNA	KX592195	DrNeurod6b	ENSDART00000018150	DrSox1a	ENSDART00000102021
CmPCNA	scaffold_5357	OlNeurod6b	ENSORLT00000022267	DrSox1b	ENSDART00000010894
LePCNA	LS-transcriptB2-ctg95554	ScNeurod2	KX592191	LoSox1b	ENSLOCT00000021941
XtPCNA	ENSXETT00000017963	CmNeurod2	XM_007901456.1	HsSox1	ENST00000330949
HsPCNA	ENST00000379143	HsNeurod2	ENST00000302584	GgSox1	AB011802.1
GgPCNA	ENSGALT00000000225	GgNeurod2	ENSGALT00000045217	PmSox3	ENSPMAT00000010751
LoPCNA	ENSLOCT00000019283	LoNeurod2	ENSLOCT00000021660		
OlPCNA	ENSORLT00000003670	OlNeurod2-1	ENSORLT00000004277		
DrPCNA	ENSDART00000076304	DrNeurod2	ENSDART00000002078		
PmPCNA	ENSPMAT00000008605	OlNeurod2	ENSORLT00000010417		
BfPCNA	XM_002588834.1				

Table S10. Accession numbers for sequences used for the phylogenies shown in Fig. S4
Novel catshark sequences were taken from Illumina transcriptomic or genomic datasets and are shown shaded in blue.

SUPPLEMENTARY REFERENCES

R Core Team (2015) R: A Language and Environment for Statistical Computing, *R Foundation for Statistical Computing*, https://www.R-project.org.

Edgar RC.(2004) MUSCLE: a multiple sequence alignment method with reduced time and space complexity. BMC Bioinformatics5:113

Kumar, S., Nei, M., Dudley, J., Tamura, K. (2008). MEGA: a biologist-centric software for evolutionary analysis of DNA and protein sequences. Brief Bioinform. *9*, 299-306.

Tippmann, H.F. (2004). Analysis for free: comparing programs for sequence analysis. Brief Bioinform. 5, 82-87.
